# High-performance mussel-inspired adhesives of reduced complexity

**DOI:** 10.1038/ncomms9663

**Published:** 2015-10-19

**Authors:** B. Kollbe Ahn, Saurabh Das, Roscoe Linstadt, Yair Kaufman, Nadine R. Martinez-Rodriguez, Razieh Mirshafian, Ellina Kesselman, Yeshayahu Talmon, Bruce H. Lipshutz, Jacob N. Israelachvili, J. Herbert Waite

**Affiliations:** 1 Marine Science Institute, University of California, Santa Barbara, California 93106, USA; 2 Chemical Engineering, University of California, Santa Barbara, California 93106, USA; 3 Chemistry and Biochemistry, University of California, Santa Barbara, California 93106, USA; 4 Molecular, Cellular and Developmental Biology, University of California, Santa Barbara, California 93106, USA; 5 Chemical Engineering, Technion-Israel Institute of Technology, Haifa 3200003, Israel

## Abstract

Despite the recent progress in and demand for wet adhesives, practical underwater adhesion remains limited or non-existent for diverse applications. Translation of mussel-inspired wet adhesion typically entails catechol functionalization of polymers and/or polyelectrolytes, and solution processing of many complex components and steps that require optimization and stabilization. Here we reduced the complexity of a wet adhesive primer to synthetic low-molecular-weight catecholic zwitterionic surfactants that show very strong adhesion (∼50 mJ m^−2^) and retain the ability to coacervate. This catecholic zwitterion adheres to diverse surfaces and self-assembles into a molecularly smooth, thin (<4 nm) and strong glue layer. The catecholic zwitterion holds particular promise as an adhesive for nanofabrication. This study significantly simplifies bio-inspired themes for wet adhesion by combining catechol with hydrophobic and electrostatic functional groups in a small molecule.

## Abstract (web summary)

Mussels use strong filaments to adhere to rocks, preventing them from being swept away in strong currents. Here, the authors borrow and simplify chemistries from the mussel foot to create a one component adhesive system which holds potential for employment in nanofabrication protocols.

## Graphical abstract

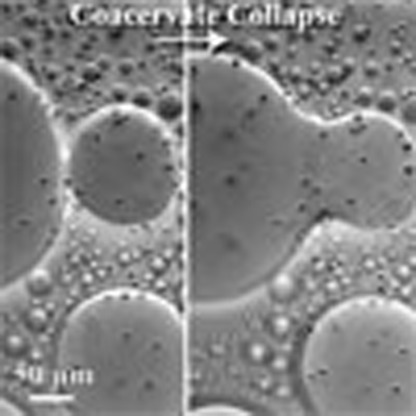

Marine mussels attach to rocks by way of thread-like tethers on wind- and wave-swept seashores where wave velocities can reach 25 m s^−1^. Mussel attachment tenacity is enabled by adaptations at multiple length scales^1^, and the nanometre scale corresponds most closely to the chemistry of the interface^2^. Interfacial mussel foot proteins (mfps), especially mfp-3 and mfp-5, are rich in the catecholic amino acid, 3,4-dihydroxyphenylalanine (Dopa), which has been adopted for functionalizing diverse synthetic polymers^3^,^4^. Although the role of Dopa in wet adhesion is under increasing scrutiny, its introduction into polymers often endows them with strong underwater adhesion^2^,^4^,^5^ and self-healing^6^,^7^,^8^ capabilities. Other common amino acids, for example, lysine, phosphoserine and histidine, are also relevant to mfp adhesion^9^,^10^ and merit translation to synthetic systems^4^,^11^. Mfps are protein polyelectrolytes with high charge densities^12^, and lend themselves to complex coacervation, which appears to be critical for the lossless fluidic delivery of mfps to target surfaces underwater^9^,^12^. Lysine and phosphoserine have been adopted to enhance adsorption^11^, but also to drive charge-dependent complexation^13^ needed for the dense fluid phase separation (coacervation) of adhesive polyelectrolytes from the equilibrium solution. Of particular interest here, mfp-5 and -3 function as adhesive primers^14^ at the plaque–substratum interface^15^. Despite recent progress in the science of wet adhesion, practical underwater adhesion remains limited or non-existent for a variety of potential applications. Biomimetic synthetic initiatives have aspired to remedy this by mimicking specific attributes of the bioadhesive model systems of mussels and sandcastle worms. These attributes include macromolecular adsorption to surfaces^2^,^3^,^16^,^17^, fluid–fluid phase separation (coacervation)^13^,^18^ and curing by oxidative catechol-based crosslinking^2^,^19^,^20^, and involve many complex components and steps requiring optimization and stabilization^21^.

In this study, both the strong wet adhesion^22^ of mfp-5 films and the coacervation^9^ of mfp-3 were translated into a smaller and simpler zwitterionic platform. We sought to simplify and combine several motifs borrowed from the interfacial adhesive mfps (including catechol, positive and negative charges, and nonpolar moieties) into a single, low-molecular-weight, one-component adhesive system. By taking a previously reported gemini zwitterionic surfactant^23^ with charged quaternary amine and phosphate groups and two alkyl tails, we replaced one of the tails with a catechol, and varied the other tail to create a family of mfp-mimetic zwitterionic adhesive homologues (mass <500 Da). These homologues exhibit very strong and spontaneous catechol-mediated binding to surfaces, stable phase separation as one-component coacervates or bilayer films, and periodate-mediated curing. The measured adhesion energy (*W*_ad_∼50 mJ m^−2^) is the highest reported to date^22^,^24^ for a nm-thick film formed underwater, and 2–3 times greater than mfp-5 (ref. 22) or the recently engineered recombinant mfp–amyloid fusion protein^24^. Zwitterion-mediated adhesion is likely to stimulate applications at multiple length scales including nanofabrications^25^,^26^ that require molecularly smooth and thin (<4 nm) adhesive layers, for example, in electronic^27^, lithographic^28^ and biomedical applications^2^.

## Results

### Zwitterionic adhesives design inspiration

**Figure 1:**
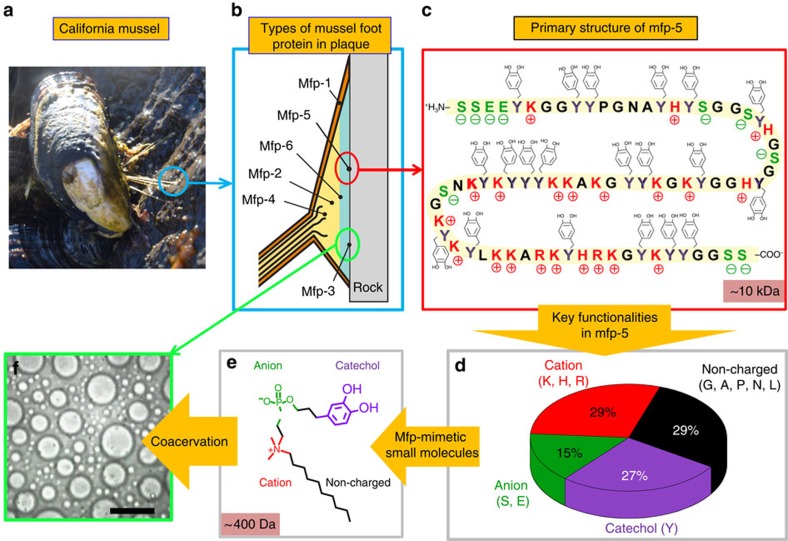
The key features of natural and translated mussel adhesion. (**a**) A mussel anchored by byssal threads and plaques to a rock in the intertidal zone (Goleta Pier, California). (**b**) Schematic of the distribution of different mfps in a plaque. (**c**) Primary sequence of mfp-5; S (green) denotes phosphoserine. (**d**) Pie chart of key functionalities in mfp-5. (**e**) One example (Z-Cat-C10) of a zwitterionic surfactant inspired by mfp-5. (**f**) Light micrograph image of liquid-phase-separated Z-Cat-C10 at 100 mg ml^−1^ concentration. The scale bar on **e** corresponds to 200 μm.

**Figure 2:**
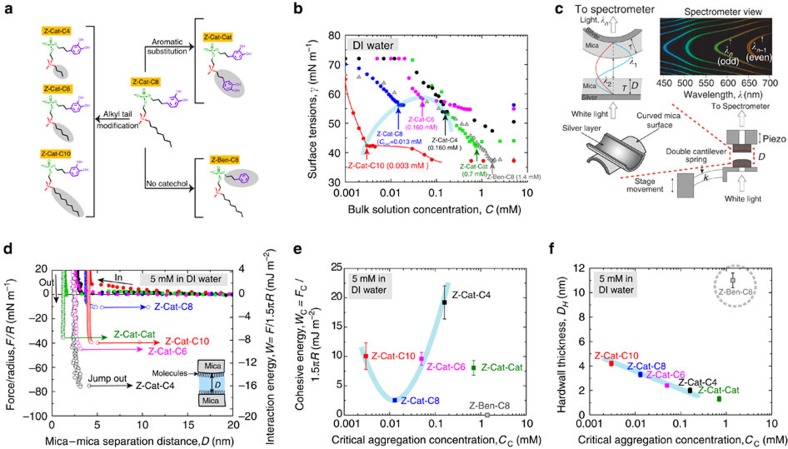
Aggregation and surface physics behaviour of mfp-mimetic zwitterions. (**a**) Summary of the engineered homologues. Features varied include alkyl chain length, aromatic substitution, with/without catechol. (**b**) CAC of each homologue is deduced from the final inflection point in the plot of log concentration versus surface tension as measured by the Wilhelmy plate technique. (**c**) Schematic of the multiple beam interferometry (MBI: the distance between the surfaces, shape of the interface and the refractive index of the media between the surfaces can be accurately determined by MBI technique) technique used in the SFA, showing the fringes of equal chromatic order used to measure the hard-wall thickness and interfacial energy of interaction between the zwitterionic films (technical details in the Supplementary Methods). (**d**) Representative force versus distance plots between mica surfaces coated with thin zwitterionic films adsorbed from 5 mM aqueous dispersions of Z-Cat-C10 (red), Z-Cat-C8 (blue), Z-Cat-C6 (pink), Z-Cat-C4 (black) and Z-Cat-Cat (green), respectively. The work of cohesion, *W*_c_ (minimum potential well of *W* versus *D*, *y* axis, right), is synonymous with the interaction energy of the zwiterions, and did not change for contact times (*t*_c_) ranging from 2 min to 12 h. Surface forces were measured during approach and separation of the surfaces, respectively. (**e**) Plot of the homologue interaction energies versus CAC. (**f**) Plot of the homologue hard-wall thickness versus CAC. Trend dashed lines (light blue) were added as a visual aid in **e** and **f**. The error bars indicate s.d.'s (*n*=5, independent experiments). DI, deionized.

The phosphate, amine, catechol and hydrophobic (alkyl or benzyl) functionalities in the zwitterions were adopted from the following mole percents of amino-acid residues in mfp-5, for example, anionic residues (∼15 mol%), cationic residues (∼29 mol%), Dopa (∼27 mol%) and assorted non-charged (∼29 mol%) (Fig. 1)^22^. Accordingly, the chemical complexity of mfps was reduced, and adhesion and coacervation were recapitulated by a strategic combination of functionalities in the zwitterionic platform (Fig. 1e,f). Various homologues were designed by replacing one or both alkyl chains with mfp-mimetic functionalities (Fig. 2a) and their interfacial and adhesive properties were investigated.

### Interfacial properties and redox stability

The aggregation tendency of each homologue in water was assessed by the critical aggregation concentration (CAC). The CAC is the concentration at which further addition of solute molecules to a solvent makes them go into finite-sized aggregates while the monomer concentration remains unchanged^29^. The CAC was determined from the solution surface tension of varying homologue concentrations in deionized water (NB the red line of Z-Cat-C10 in Fig. 2b) by Wilhelmy plate tensiometry. Figure 2b shows that decreasing alkyl tail length increases the CAC gradually from Z-Cat-C10 to -C8, -C6 and -C4 (parabolic dashed line drawn in light blue from Z-Cat-C10 to -C4), consistent with thermodynamic predictions^29^. Conversely, aromatic substitution (Fig. 2a) increased CAC significantly. The substitution of the catechol of Z-Cat-C8 by a benzene (Fig. 2a) also increased CAC. On the basis of cyclic voltammetry combined with ultraviolet–visible spectroscopy, the oxidative stability of catechol functionalities in all zwitterionic homologues (for example, *E*_½_ of Z-Cat-C10∼+0.25 V at 1 mV s^−^1) was significantly increased by comparison with 4-methylcatechol (completely solvent accessible, Δ*E*_½_∼+0.12 V in deionized water at 1 mV s^−^1), suggesting a highly stabilizing environment in the dispersion (Supplementary Fig. 1).

### Surface force measurements and interfacial interaction study

Thin films of the zwitterionic homologues were deposited on mica surfaces by adsorption from 5 mM solution (>CAC for all homologues; for concentrations below CAC see Supplementary Discussion), and investigated by the surface forces apparatus (SFA; Fig. 2c), atomic force microscopy (AFM; Supplementary Figs 4, 17 and 18) and quartz crystal microbalance with dissipation (QCM-D; Supplementary Figs 5 and 6). Surface force measurements between symmetric thin films on mica were investigated in the SFA. On approach of the two surfaces, Z-Ben-C8 films showed regular bilayer repulsion and jump-in instabilities typical for surfactant layers (Supplementary Fig. 8), whereas the catecholic homologues showed only slight or no repulsion (Fig. 2d). Z-Cat-C4 (19.2±2.4 mJ m^−2^) exhibited the greatest cohesion (or adhesion) energies in deionized water, followed by Z-Cat-C10 (10.1±2.3 mJ m^−2^), Z-Cat-C6 (9.6±1.1 mJ m^−2^), Z-Cat-Cat (8.1±1.3 mJ m^−2^) and Z-Cat-C8 (2.5±0.3 mJ m^−2^) (Fig. 2e). Notably, the interaction energies of Z-Cat-C10, -C8, -C6 and -C4 were closely correlated with the CAC (light blue parabola drawn in the plot in Fig. 2e as in Fig. 2b).

The hard-wall thickness (the limiting distance between the mica surfaces during the approach run in the SFA) of each homologue was measured and correlated with the CAC (Fig. 2f) corresponding to the catechol-containing zwitterions, which formed strongly cohesive (*W*_c_=2.2–21.6 mJ m^−2^) bilayers (0.5–2.0 nm). In contrast, the non-catecholic Z-Ben-C8 formed multilayered films (∼5 nm) without cohesive tendencies. In the catechol-containing homologues with alkyl tails (Fig. 2a), for example, Z-Cat-C10, -C8, -C6 and -C4, the hard-wall thickness and CAC were both correlated with alkyl tail length (light blue line in Fig. 2f).

**Figure 3:**
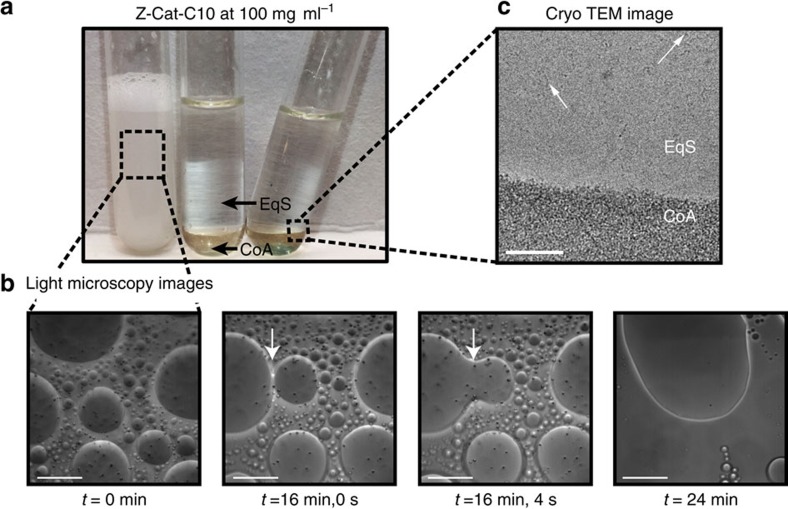
Fluid-fluid phase separation of mfp-mimetic zwitterions. (**a**) Phase separation of Z-Cat-C10 (100 mg ml^−1^) in 4-mm-diameter glass test tubes. Shown are the turbid dispersions of coacervate microdroplets (left) and the bulk-separated phases with the denser coalesced coacervate phase on the bottom (middle) and tilted to emphasize the fluidity of both phases (right). (**b**) Light micrographs showing the time course of coacervate coalescence in Z-Cat-C10 at 0–24 min. The 16 min, 4 s image shows coalescence of two microdroplets. Scale bar, 100 μm. (**c**) Cryo-TEM image of the dense phase; the image of turbid dispersion is similar. White arrows indicate same small aggregations in both equilibrium solution (EqS) and coacervate phase (CoA), an area of a large aggregate of those smaller aggregates seen in the lower part of the field of view. Scale bar, 100 nm.

Z-Cat-C10, which exhibited the lowest CAC among the homologues and readily phase separated into dense visible coacervates and equilibrium solution at high concentrations (100 mg ml^−1^), was chosen for further scrutiny (Fig. 3a). Coalescence of the coacervate microdroplets in bulk solution was observed by microscopy (Fig. 3b and Supplementary Movie 1), and spreading of coacervates over a glass substrate was recorded on an inverted microscope (Supplementary Movie 2). Cryogenic transmission electron microscopy (cryo-TEM) images (Fig. 3c and Supplementary Figs 13 and 14) showed small micelle-like aggregates in both equilibrium solution and coacervate phases.

**Figure 4:**
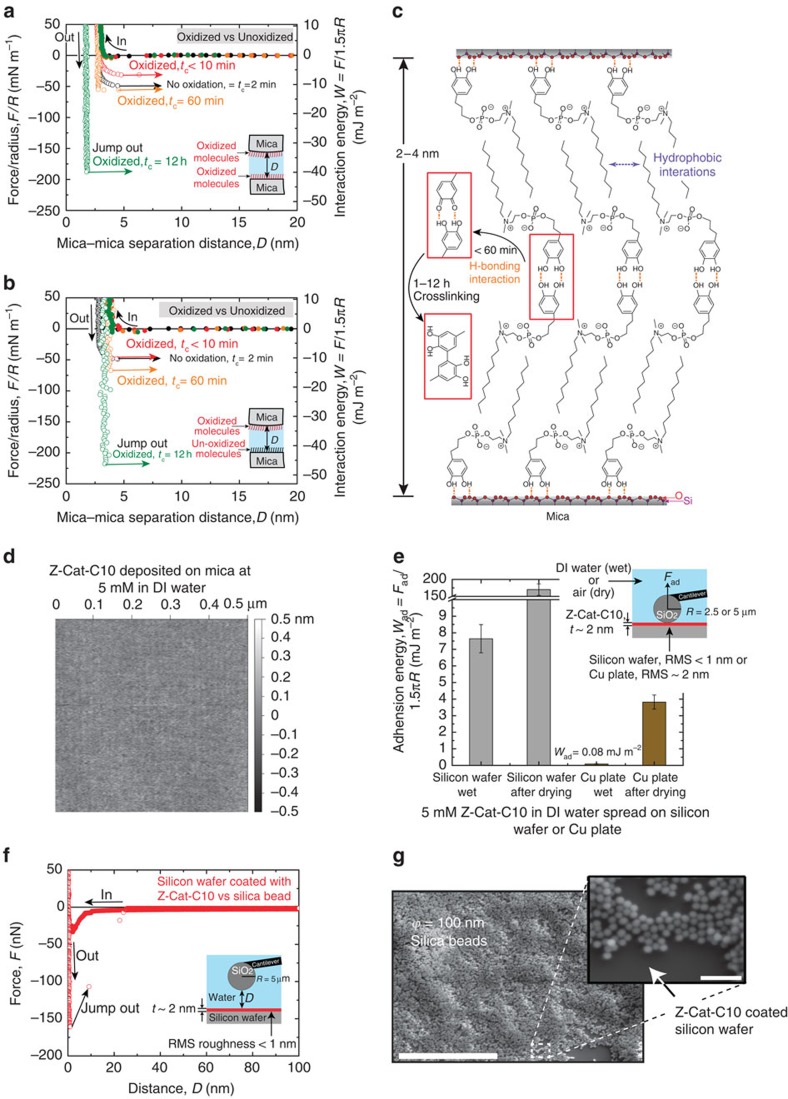
Atomically smooth and thin nano-adhesives from mfp-mimetic zwitterion. (**a**,**b**) Representative force versus (vs) distance plots of symmetric (**a**) and asymmetric (**b**) periodate oxidized Z-Cat-C10 films on mica. Before periodate, the work of adhesion *W*_ad_ did not change for contact times (*t*_c_) from 2 min to 12 h (black). After oxidation with periodate (250 pmoles), *t*_c_≤10 min (red points), *t*_c_=60 min (orange) and *t*_c_=12 h (green). Filled and open circles represent the forces measured during approach and separation of the surfaces, respectively. (**c**) Cartoon of the two self-assembled nano-glue layers (upper and lower, respectively) and the interface. (**d**) AFM image of atomically smooth adhesive layer formed from 5 mM Z-Cat-C10 on mica (the layer on SiO_2_ and CuO_2_/Cu(OH)_2_ shown in Supplementary Figs 15 and 16, respectively). (**e**) Adhesion energy (*W*_ad_=*F*_ad_/1.5π*R*) of the nano-glue from Z-Cat-C10 measured on silicon wafer and copper plate, where adhesion force *F*_ad_ (minimum of the potential well of the *F* versus *D* curves obtained from AFM measurements) and *R* (radius of silicon dioxide probe). (**f**) Representative force versus distance plots of the nano-glue from 5 mM Z-Cat-C10 in AFM. (**g**) SEM image of underwater glued nano silica beads (*φ*=100 nm) on silicon wafer by the nano-glue from 5 mM Z-Cat-C10. The error bars indicate s.d.'s (*n*=5, independent experiments). Scale bar, 500 μm; inset scale bar, 500 nm. DI, deionized.

Mussels rely on quinone-mediated protein crosslinking to increase plaque cohesion. o-Quinones are produced by the two-electron oxidation of targeted Dopa residues^22^. Accordingly, we oxidized the catechol in Z-Cat-C10 with sodium periodate resulting in a stoichiometric −2H^+^ and −2e^−^ oxidation to o-quinone^19^. Given the nanoNewton precision of the SFA, we measured the adhesion of monomolecular layers of Z-Cat-C10 on mica. SFA force–distance profiles between surfaces allow the measurement of adhesion forces that can be translated into adhesion energies (*W*_ad_) by the Johnson–Kendall–Roberts theory for soft deformable surfaces^29^,^30^. Analysis by SFA showed that the observed cohesion or adhesion energy correlated well with the symmetric or asymmetric oxidation of catechol by periodate^7^. After symmetric periodate oxidation (Fig. 4a), cohesion initially decreased by 50% for contact times of ≤*t*_c_=10 min, but then rebounded after *t*_c_=60-min contact times. Conversely, asymmetric oxidation, that is, all catechol on one side and all quinone on the other (Fig. 4b), maintained the initial adhesion for contact times of ≤*t*_c_=10 min, followed by a 50% increase in adhesion after a *t*_c_=60-min contact time. The loss^22^,^24^ and subsequent recovery^22^ of adhesion, respectively, following oxidation were previously explained as the loss of H-bonding interactions because quinones are exclusively H-bond acceptors (short term), followed by the quinone-mediated formation of covalent crosslinks (long term). The high adhesion (*W*_ad_∼50 mJ m^−2^) obtained after *t*_c_=12 h of compressive contact, contrasts with ≤*t*_c_=10 min and *t*_c_=1 h contacts. According to the post-oxidation analyses, covalent crosslinks formed within and between the thin films (∼2–4 nm) (Fig. 4c).

Not limited to mica (Fig. 4d), 5 mM Z-Cat-C10 formed thin, molecularly smooth (∼2 nm) films on silicon wafers (Supplementary Fig. 15) and copper (Supplementary Fig. 16). The wet and dry adhesion of these films (Supplementary Figs 17–20) on silicon wafers (SiO_2_, root mean squared (r.m.s.) surface roughness <1 nm) and copper (CuO_2_/Cu(OH)_2_, r.m.s. surface roughness ∼2 nm) surfaces was measured using silica (SiO_2_) probes in the AFM (Fig. 4e). The surfaces were prepared by spreading 5 mM Z-Cat-C10 over the surfaces, followed by thorough rinsing and adhesive testing (Fig. 4f). The wet work of adhesion (*W*_ad_=*F*_ad_/1.5*πR*), ∼7.7  mJ m^−2^ for the SiO_2_ and SiO_2_ interface, resembled *W*_ad_ ∼7.0 mJ m^−2^ reported for catechol-functionalized polysiloxanes and SiO_2_ interface^31^, and ∼0.1 mJ m^−2^ for adhesion between the CuO_2_/Cu(OH)_2_ and SiO_2_ surfaces. After air drying the surfaces for ∼5 min, the adhesion (*W*_ad_) between CuO_2_/Cu(OH)_2_ and SiO_2_ surfaces increased significantly to ∼4 mJ m^−2^ and to ∼175 mJ m^−2^ between SiO_2_ and SiO_2_ surfaces, which, in the latter case, is 50-fold greater than the *W*_ad_ (∼3.5 mJ m^−2^) for catecholic polysiloxane adhesion to SiO_2_ as calculated from the *F*_ad_ (83 nN) measured using silica probes (*φ*=5 μm) in AFM^31^. Comparatively weaker adhesion to the copper plate is tentatively attributed to higher r.m.s. and hydroxides on its surface measured by AFM and X-ray photoelectron spectroscopy (XPS), respectively (Supplementary Figs 21–26). We also prepared a suspension of silica beads (*φ*=100 nm) in deionized water, and spread it over a silicon wafer previously coated with 5 mM Z-Cat-C10. The silica beads remained glued to the film, even after rinsing with pulsed jets of running tap water (0.24 MPa; Fig. 4g).

## Discussion

Catechol-functionalized zwitterionic surfactants are clearly more than detergents, and show the following favourable attributes as adhesive primers: (1) they aggregate in water as dense fluidic coacervates at concentrations above the CAC; (2) the coacervates spread uniformly over surfaces; (3) in confinement in the SFA, the coacervates rearrange as paired bilayers (∼2.5-nm thickness) with the catecholic head groups facing the mica surface on one side and bulk solvent on the other; (4) the coacervate environment stabilizes catechols by ∼+0.1 V against oxidation; and (5) following periodate treatment, adhesion of thin films of Z-Cat-C10 on mica approaches *W*_ad_∼50 mJ m^−2^—a new high value for catechol-mediated adhesion.

The results reveal Z-Cat-C10 adhesion on mica to be two- to three-fold greater than mfp-5 (14 mJ m^−2^) (ref. 22) and the new mfp–amyloid recombinant fusion protein (21 mJ m^−2^) (ref. 24). Results obtained before and after periodate oxidation of Z-Cat-C10 suggest the following failure modes: before periodate, the work of adhesion averaged ∼20 mJ m^−2^. Because the hydrophobic bilayer and catechol-to-mica contacts are strong, the weaker inter-catechol H-bonding between the two bilayers (Fig. 4c) unzips during separation. After covalent aryl–aryl coupling with periodate, the adhesion failed at ∼50 mJ m^−2^, which is commensurate with the energy required to separate the two halves of a lipid bilayer leaflet^32^, thus suggesting that adhesion to the mica surface is stronger than ∼50 mJ m^−2^.

Because the SFA-based work of adhesion (units J m^−2^) is difficult to relate to reported bulk adhesion properties (J m^−3^), we shall compare it with peel fracture energy. With native mussel plaques deposited onto silica, peel fracture energy^1^ was 100 J m^−2^, which resembles the energy of high-performance adhesive polystyrene; polyvinylpyrrolidone (PS/PVP) blends on silicon^28^. The magnitude of disparity between the work of adhesion and peel fracture energy is striking but plausible. In tests of symmetrically deposited Z-Cat-C10, the paired bilayer structure (Fig. 4c) offers little opportunity for energy dissipation via sacrificial bonds and hidden lengths^33^, whereas in the plaque, molecular scaffolds of different length scales exist^34^. The relationships between the adhesive surface primers and their overlying molecular scaffolds are not well understood. Although the catechol-functionalized zwitterionic surfactants were designed to be adhesive primers, some bulk tests in lap shear or a three-point bending peel mode on stainless steel (SS304) (Supplementary Fig. 12) showed unexpected gap-filling capabilities and strengths of ∼1 MPa or ∼20 N cm^−1^, suggesting that stacked bilayers as shown in Fig. 4c may not be limited to two layers.

In summary, we show that a synthetic low-molecular-weight catecholic zwitterionic surfactant mimics the strong adhesive priming and self-coacervating properties of mfps. The high wet-cohesion (or -adhesion) energies ranged from ∼20 to ∼50 mJ m^−2^ for uncrosslinked and crosslinked films, respectively. This study underscores the importance of catechols and of maintaining a balance^4^ between hydrophobic and electrostatic interactions for tuning or optimizing both coacervation and adhesion. These catecholic zwitterions are adaptable as coatings or adhesive primers^14^,^28^ for diverse surfaces and, given their uniformly thin (<4 nm) and strong glue layers, hold particular promise as a new tool for nanofabrication^25^,^26^,^27^,^35^ (please see Supplementary Note and Discussion for appropriate details).

## Methods

### Synthesis

The zwitterionic surfactants were synthesized based on a previously described procedure^23^ (Supplementary Methods).

### Critical aggregation concentration

Aqueous colloidal dispersions of each homologue were prepared in deoxygenated deionized water. The CAC was determined from the solution surface tension of varying homologue concentrations in deionized water (for example, see the red line of Z-Cat-C10 in Fig. 2b) by Wilhelmy plate tensiometry and has been described elsewhere^36^. Use of the CAC instead of Critical Micelle Concentration (CMC) here is a hedge to accommodate our uncertainty about whether micelles or other soluble aggregates were forming.

### Cyclic voltammetry

Cyclic voltammetry (CV) was performed on a Versastat 3 potentiostat from Ametek Co. (Berwyn, PA). These analyses were carried out using a three-electrode cell: a Pt wire as the counter electrode; an Ag/AgCl reference electrode; and a carbon paste electrode (CPE) served as the working electrode. Carbon paste electrode is the best choice for adhesive materials because of its ability to be completely polished before each experiment.

### Measuring adhesion and cohesion energies

The adhesion/cohesion energy of the synthetic zwitterionic molecules was investigated in SFA (SurForce LLC, Santa Barbara, CA). Two molecularly smooth freshly cleaved back-silvered mica surfaces glued on cylindrical silica discs of radius of curvature, *R*∼2 cm were mounted in the SFA and 50 μl of a colloidal dispersion of the synthetic molecules in deionized water was injected between the surfaces at different concentrations (*C*=0.5–5 mM) and the forces between the thin films of the molecules were measured by multiple beam interferometry^37^. The adhesion forces between Z-Cat-C10 molecules on various surfaces, for example, silica and copper, were also measured by AFM (MFP-3D, Asylum Research, Goleta, CA) in deionized water and ambient conditions, respectively, at room temperature. AFM probes with a silicon dioxide particle (10, 5 and 1 μm, respectively) on a silicon nitride (SN) cantilever −0.6 N m^−1^ were purchased from Novascan Technolgies, Inc. (Ames, IA). Cantilever spring constants were measured for each force run. Images of Z-Cat-C10 coating on silicon wafer and copper plates were visualized in an AFM.

### Cryo-TEM

Cryo-TEM vitrified specimens were prepared in a controlled environment vitrification system at 25 °C and 100% relative humidity. The procedure is described in detail elsewhere^38^. About 3 μl of the sample was applied onto a perforated (lacey) carbon film-coated 200 mesh copper TEM grid. Excess liquid was blotted with filter paper, and the specimen was immediately plunged into liquid ethane at its freezing point (−183 °C). The vitrified specimens were 240 transferred to a 626 Gatan cryo-holder, using it tansfer station, and imaged at 120 kV acceleration voltage in an FEI Tecnai T12 G^2^ TEM, using low-dose imaging to minimize electron-beam radiation damage. Typical electron exposures were 10 to 15 e^−^ Å^−2^. The specimens were kept in the TEM at about −175 °C. Images were digitally recorded with a Gatan US1000 high-resolution (2it × 2high-resolution (2charge-coupled device camera, using the Digital Micrograph software.

### QCM-D

A ‘static cell' (often called ‘Open Model') QCM-D (Q-Sense, Biolin Scientific, Linthicum Heights, MD) was employed to qualitatively show the adsorption of the zwitterionic molecules onto mineral and metal oxide surfaces (Supplementary Methods).

### XPS

XPS (Kratos Axis Ultra, Kratos Analytical, Manchester UK) was conducted with survey scans at a pass energy of 160 eV, and high-resolution scans at a pass energy of 40 eV. Nano-layer adhesion to silica surface was imaged with SEM after rinsing silica bead (diameter *ϕ*=100 nm) dispersion (10 mg ml^−1^ in deionized water), spread on a silicon wafer coated with Z-Cat-C10, thoroughly with deionized water.

## Additional information

**How to cite this article:** Ahn, B. K. *et al*. High-performance mussel-inspired adhesives of reduced complexity. *Nat. Commun*. 6:8663 doi: 10.1038/ncomms9663 (2015).

## Supplementary Material

Supplementary Information Supplementary Figures 1-28, Supplementary Notes 1-6, Supplementary Discussion, Supplementary Methods and Supplementary References

Supplementary Movie 1 Coalescence of the coacervate microdroplets in bulk solution.

Supplementary Movie 2 Spreading of coacervates over a glass substrate recorded on an inverted microscope.

Supplementary Movie 3 Lap joint test- Gluing two steel plates (SS304) immersed in water using the dense coacervate (~10 μl) of Z-Cat-C10
