# Supplementary material for: High-performance mussel-inspired adhesives of reduced complexity
Source: Nat Commun. 2015 Oct 19;6:8663. doi: 10.1038/ncomms9663 (PMC4667698; doi:10.1038/ncomms9663)
Supplement: Supplementary Information — Supplementary Figures 1-28, Supplementary Notes 1-6, Supplementary Discussion, Supplementary Methods and Supplementary References [file ncomms9663-s1.pdf]

Supplementary Figures

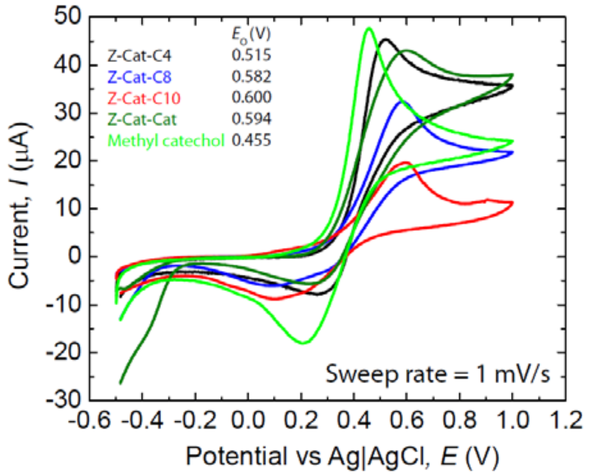

Supplementary Figure 1. Cyclic voltammetry measurements for 5 mM solution of the small molecules in DI water.

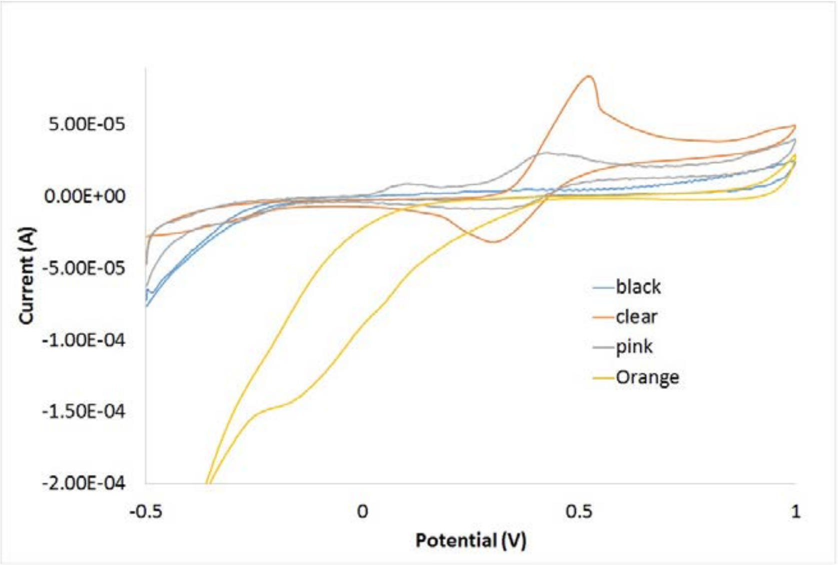

Supplementary Figure 2. Cyclic Voltammetry (CV) of the Z-Cat-C10 coacervate injected into DI water (clear solution), artificial seawater (pink solution), periodate in DI water (orange solution), and steal plate in artificial seawater (black solution).

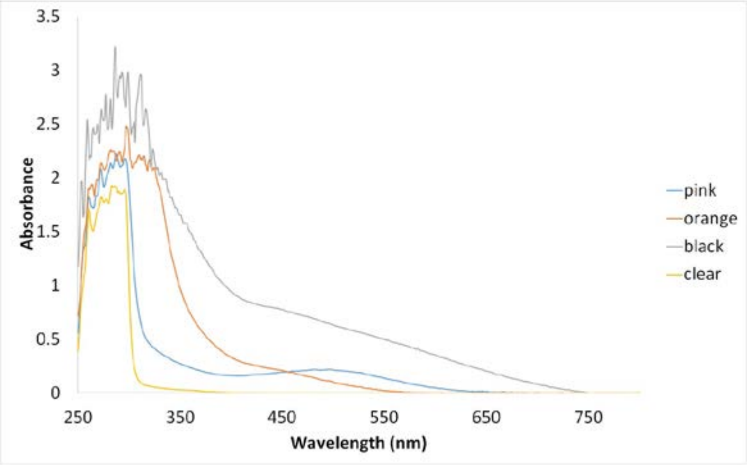

Supplementary Figure 3. UV-Vis of the Z-Cat-C10 coacervate injected into DI water (clear solution), artificial sea water (pink solution), periodate in DI water (orange solution), and steal plate in artificial sea water (black solution).

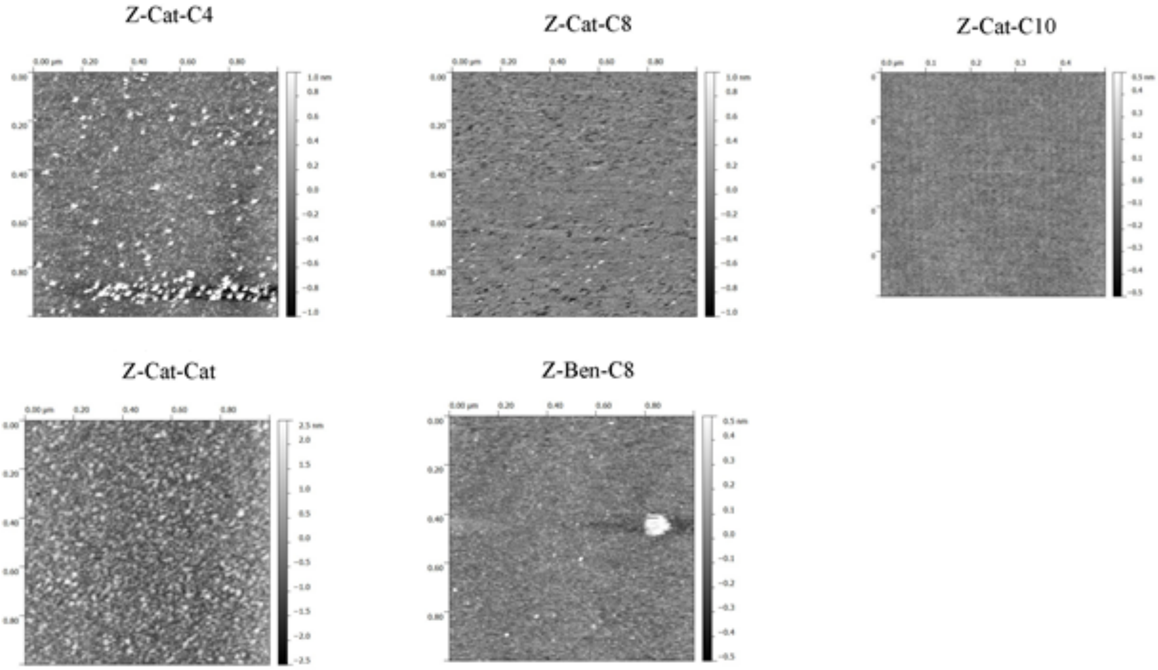

Supplementary Figure 4. AFM images of the small molecules absorbed on mica surfaces.

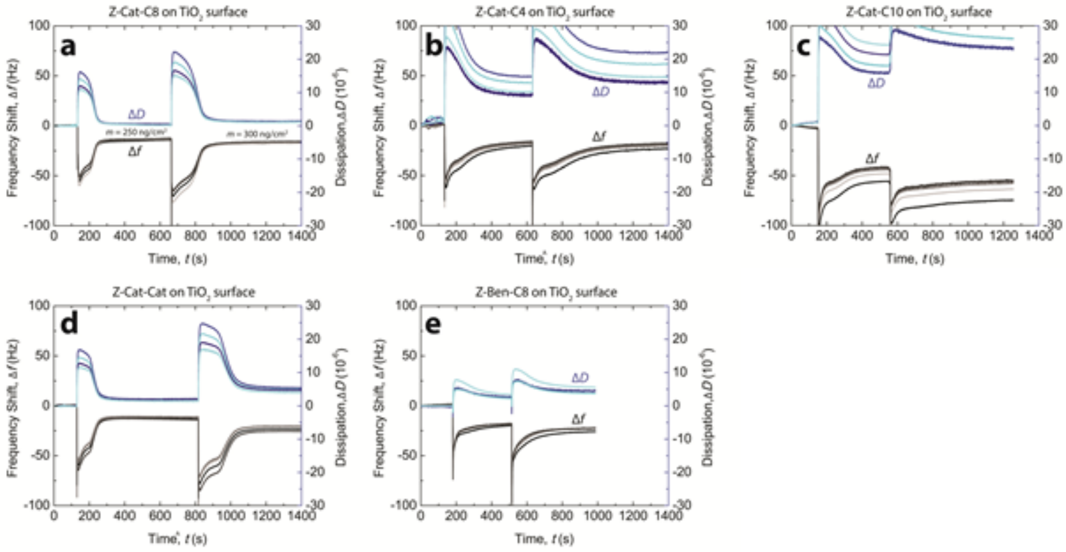

Supplementary Figure 5. QCM-D experiments showing the adsorption of all molecules onto a TiO_2 surface. Frequency and dissipation change upon addition of (5 mM) of each single molecule solution to a titania surface. In this two-step adsorption, each molecule was deposited (50 µL) onto a titania surface in DI H_2O using a static cell.

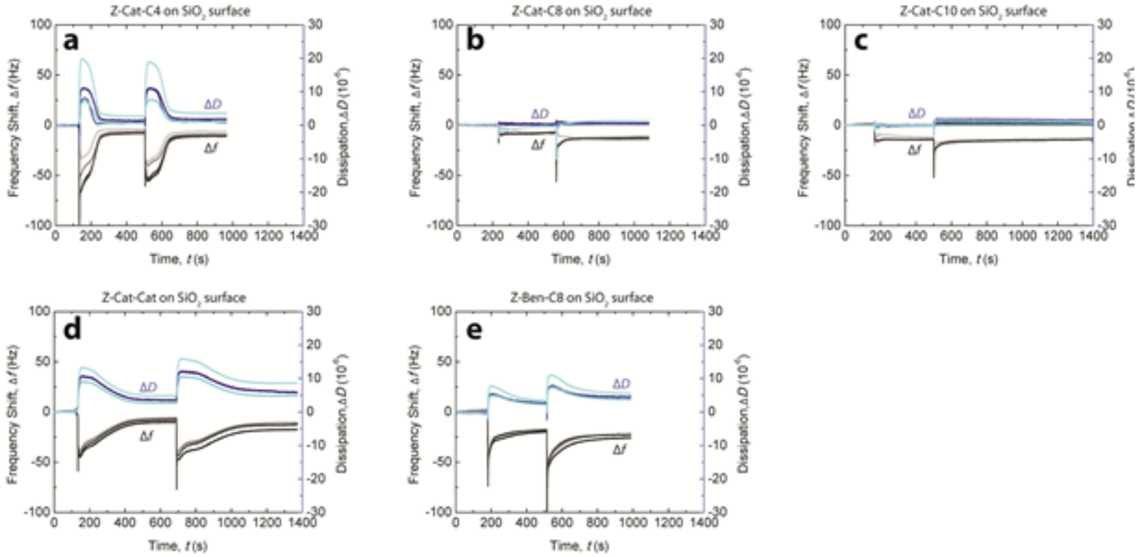

Supplementary Figure 6. QCM-D experiments showing the adsorption of all molecules onto a SiO_2 surface. Frequency and dissipation change upon addition of (5 mM) of each single molecule

solution to a silica surface. In this two-step adsorption, each molecule was deposited (50 μL) onto a silica surface in DI H_2O using a static cell.

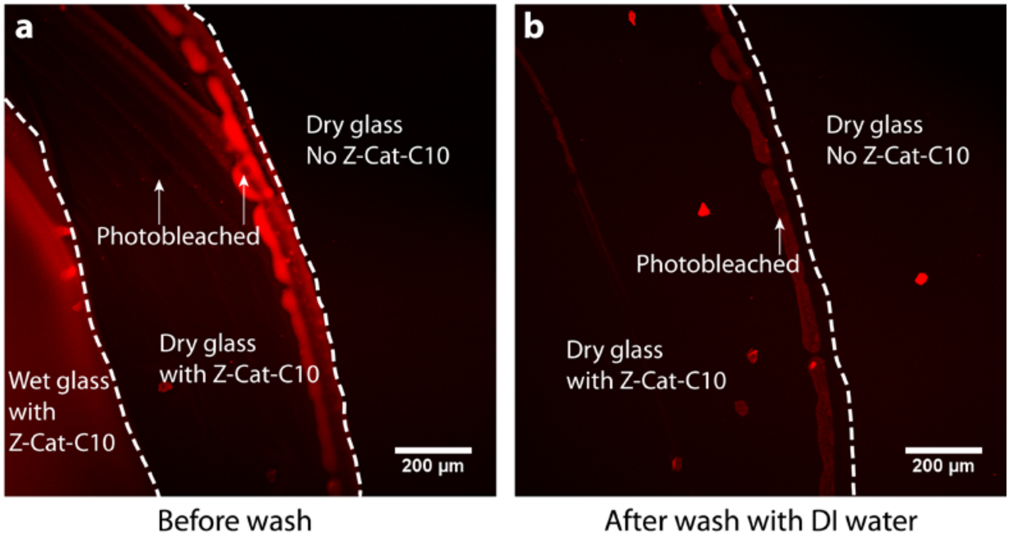

Supplementary Figure 7. Fluorescence imaging of a silica surface coated with a 5 mM solution of Z-Cat-C10 (in DI water) before and after rinsing the surface with DI water.

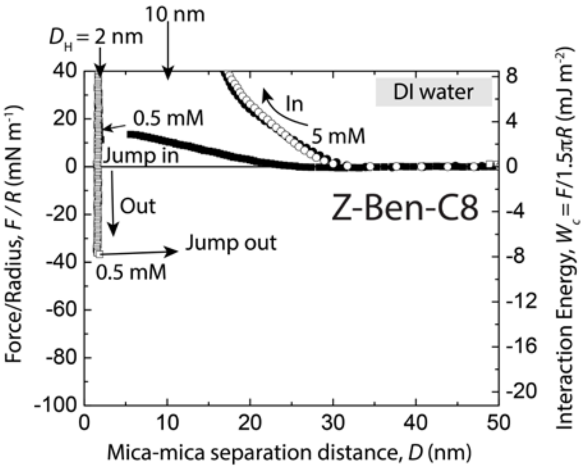

Supplementary Figure 8. The representative force *vs.* distance plots between mica surfaces of 0.5 mM and 5 mM aqueous despersions of Z-Ben-C8.

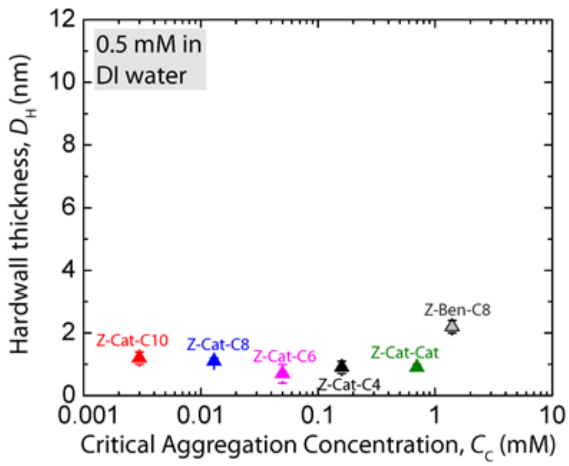

Supplementary Figure 9. Plot of the hardwall thickness in 0.5 mM concentration *vs.* CAC of Z-Cat-C10 (red points), -C8 (blue points), -C6 (pink points), -C4 (black points), and -Cat-Cat (green points), respectively. Notice that error bars indicate standard deviation (n=5 independent experiment).

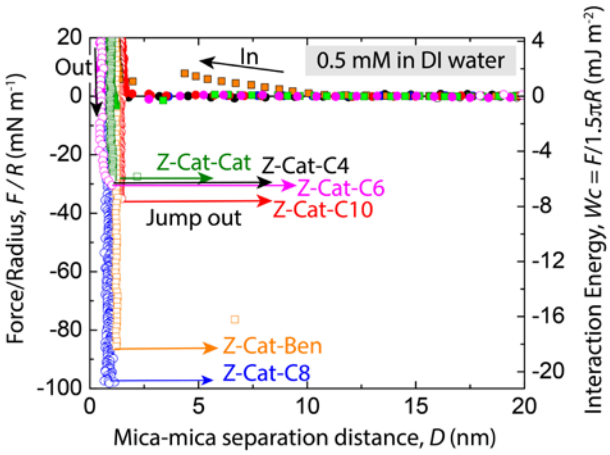

Supplementary Figure 10. The representative force *vs.* distance plots between mica surfaces of 0.5 mM aqueous despersions of Z-Cat-C10 (red points), -C8 (blue points), -C6 (pink points), -C4 (black points), and -Cat-Cat (green points), respectively.

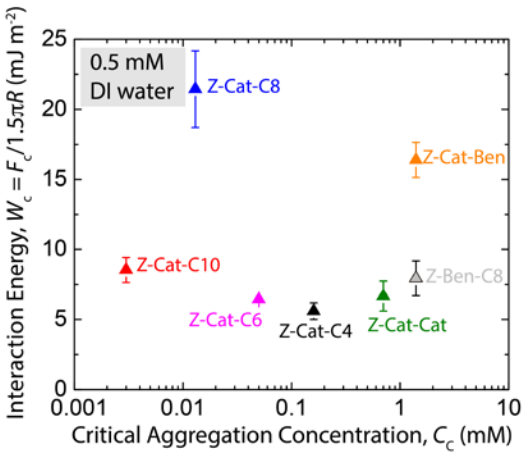

Supplementary Figure 11. Plot of the adhesive interaction energy *vs.* the CAC. Notice that error bars indicate standard deviation (n=5 independent experiment).

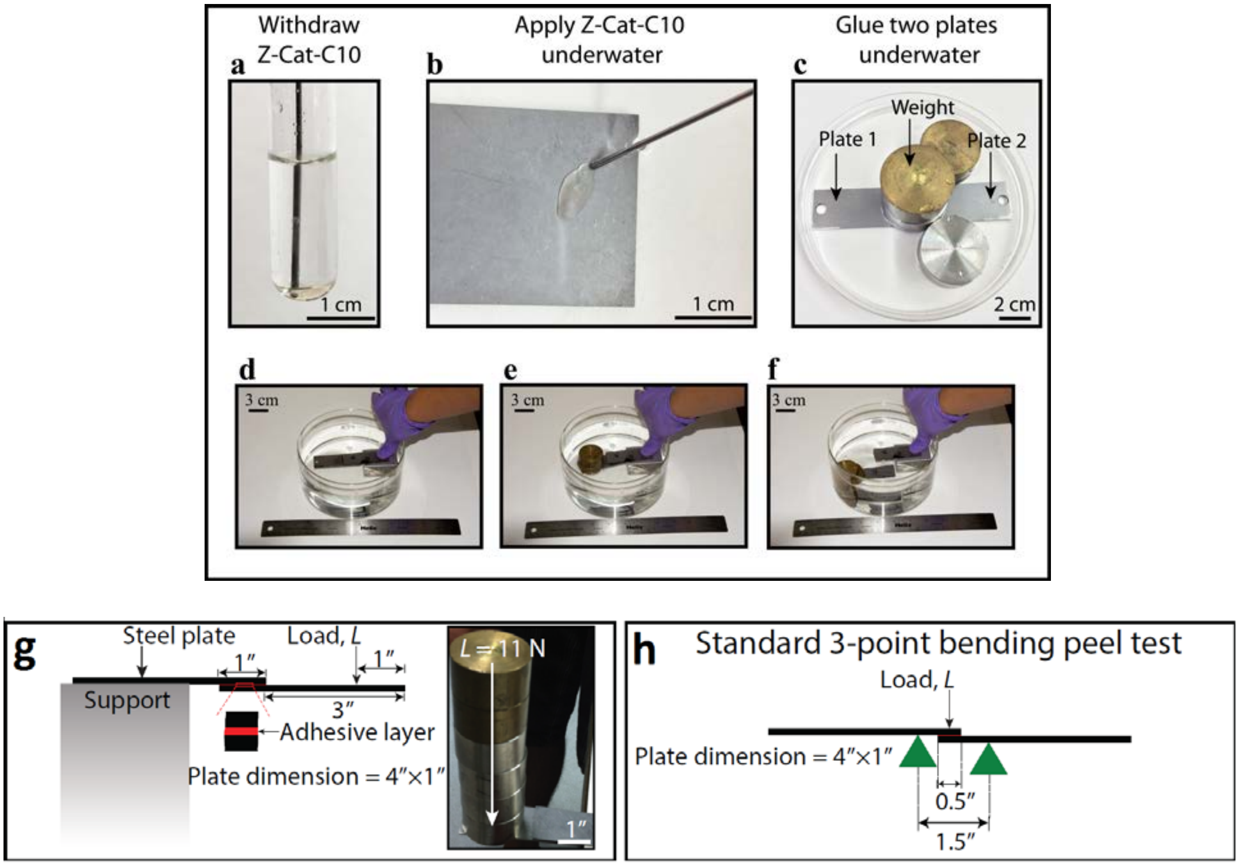

Supplementary Figure 12. A macro-scale lab adhesion test: the phase-separated fluid at the

bottom was collected with a syringe (a) and injected on to a top of steal plated underwater (b), then glued onto the other plate (c), and the lab adhesion test (d, e, and f) after 12h. (g) Demonstration of lap joint fracture test (apparent cross section = 2.54 ×2.54 cm). (h) Demonstration of standard 3 point bending peel test (apparent cross section = 1.27 ×2.54 cm).

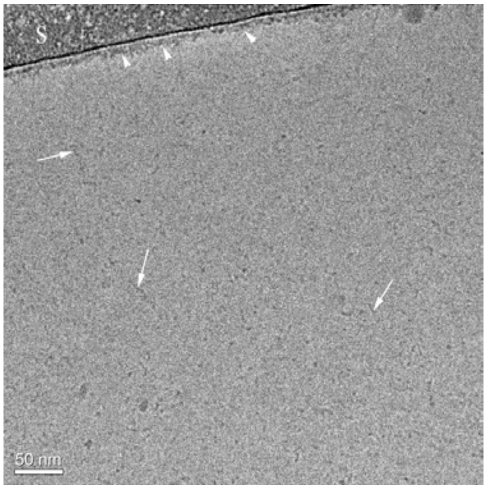

Supplementary Figure 13. Cryo-TEM image shows mostly small aggregates (arrows) and some individual aggregates (arrowheads) attached to the support film (S).

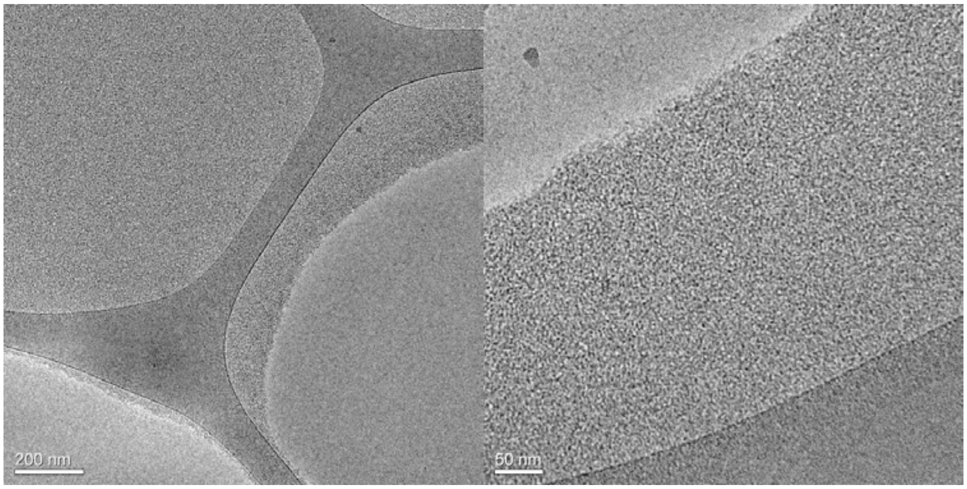

Supplementary Figure 14. Cryo-TEM images show large domains of packed aggregates attached to the carbon support film.

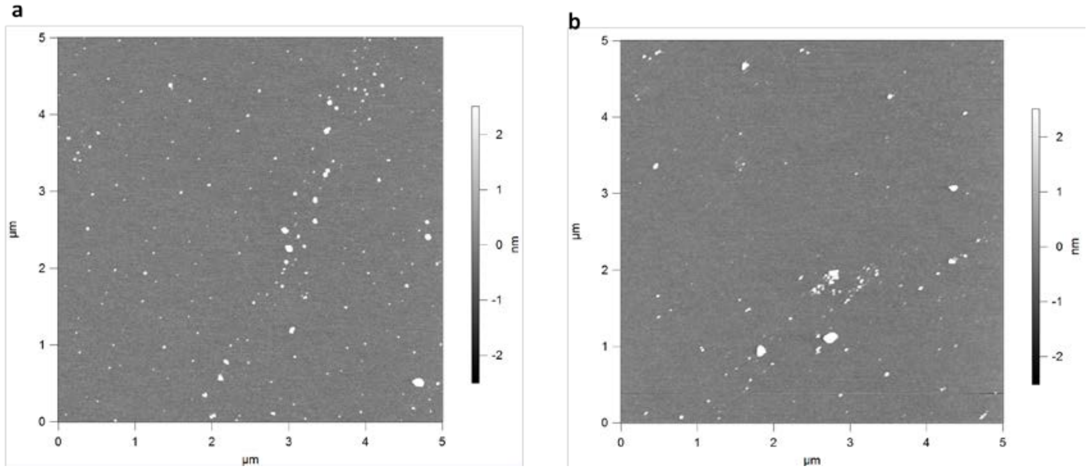

Supplementary Figure 15. AFM images of a silicon wafer: (**a**) a plane SiO_2 surface (RMS ~ 1 nm), (**b**) a SiO_2 surface coated with Z-Cat-C10 (RMS ~ 1 nm): subsequently after spreading the 5 mM Z-Cat-C10, waiting for 2min, rinsing thoroughly, and drying for ~5min.

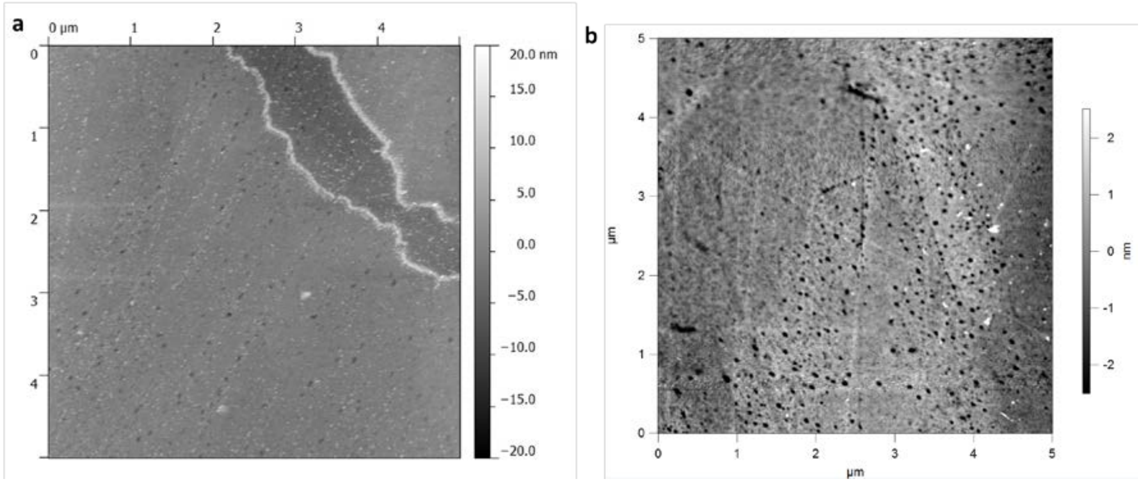

Supplementary Figure 16. AFM images of a copper plate: (**a**) a plane $CuO_2/Cu(OH)_2$ surface (RMS ~ 2 nm), (**b**) a $CuO_2/Cu(OH)_2$ surface coated with Z-Cat-C10 (RMS ~ 2 nm): subsequently after spreading the 5 mM Z-Cat-C10, waiting for 2min, rinsing thoroughly, and drying for ~5min.

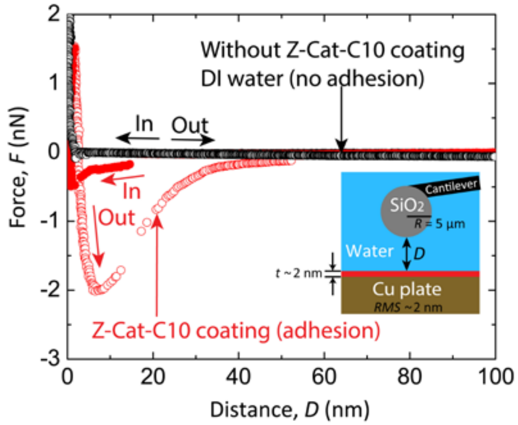

Supplementary Figure 17. AFM force run on copper plate underwater using ϕ=10 µm silica tip. Cantilever spring constant (K) was 0.1 N m^{-1}.

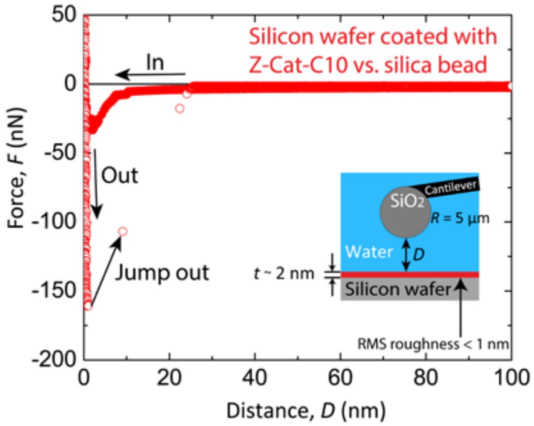

Supplementary Figure 18. AFM force run on silicon wafer underwater using ϕ=10 µm silica tip. Cantilever spring constant (K) was 0.1 N m^{-1}.

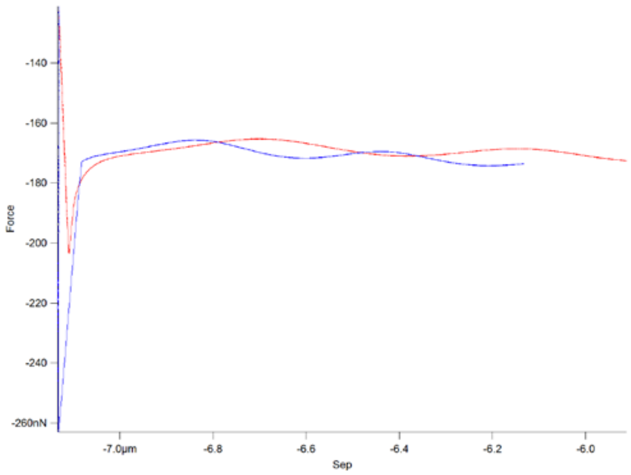

Supplementary Figure 19. AFM force run on copper plate in ambient condition using ϕ=5 µm silica tip after drying the substrate for ~5 min. Cantilever spring constant (K) was 1.8 N m^{-1}. Force measured in red when approaching and in blue when separating.

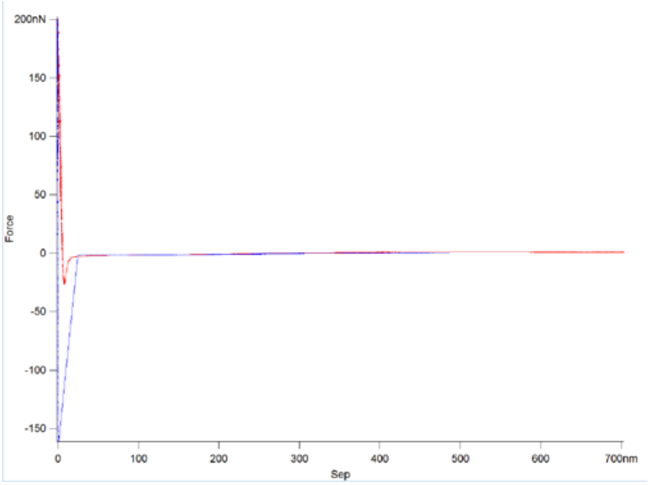

Supplementary Figure 20. AFM force run on silicon wafer in ambient condition using ϕ=1 µm silica tip after drying the substrate for ~5 min. Cantilever spring constant (K) was 6 N m^{-1}. Force measured in red when approaching and in blue when separating.

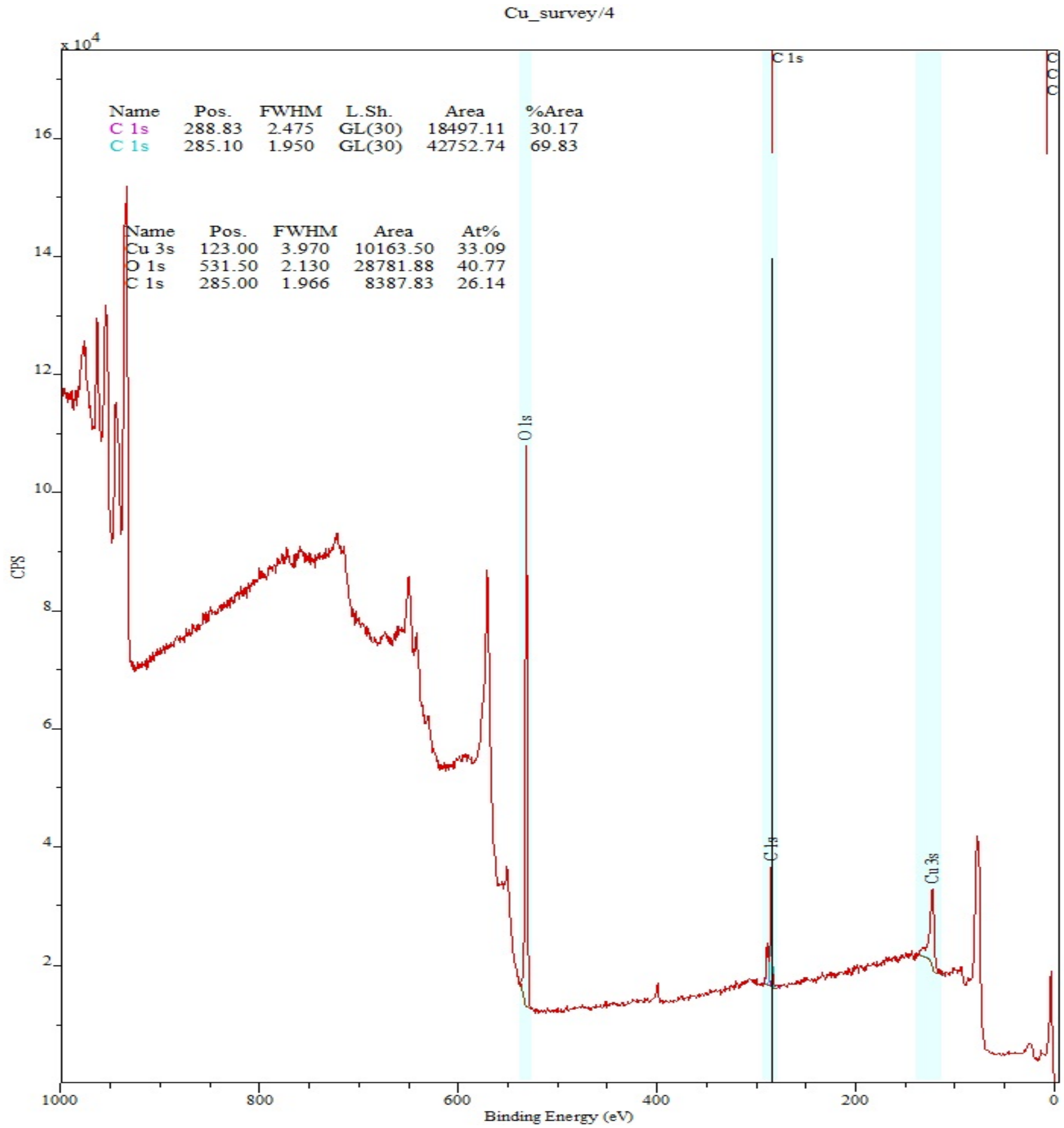

Supplementary Figure 21. XPS of copper plate survey.

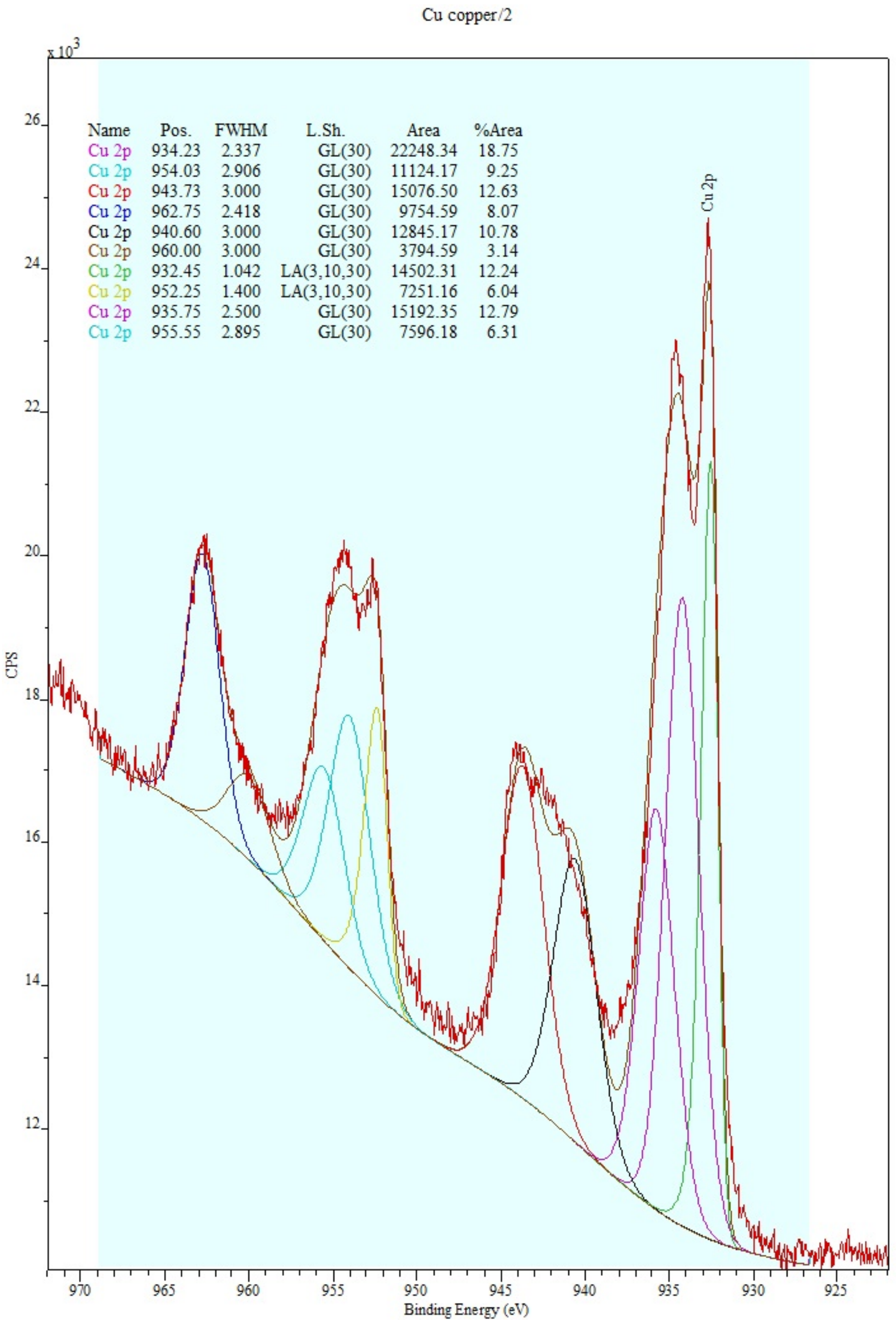

Supplementary Figure 22. XPS of copper plate, high resolution Cu 2p.

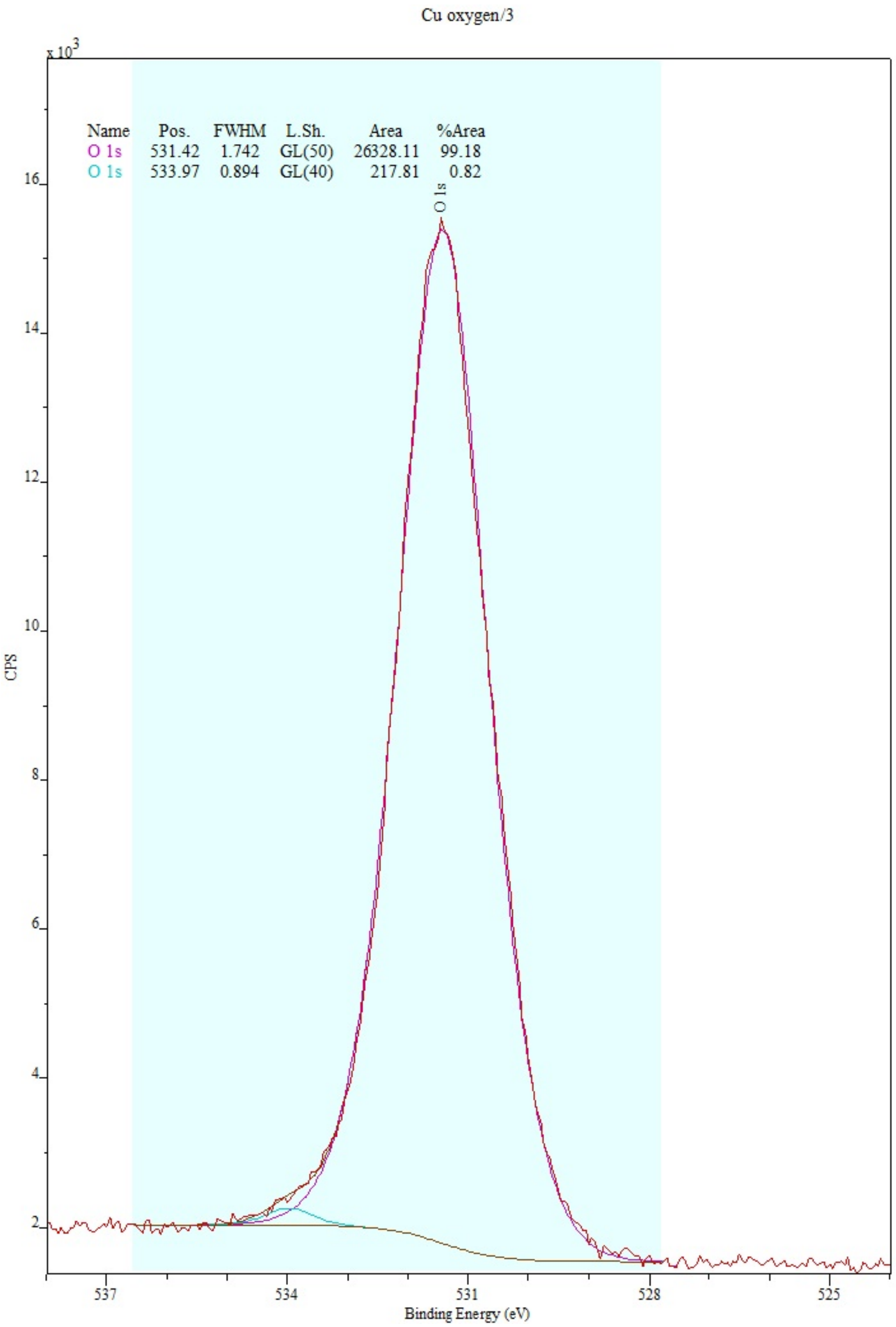

Supplementary Figure 23. XPS of copper plate, high resolution O1s.

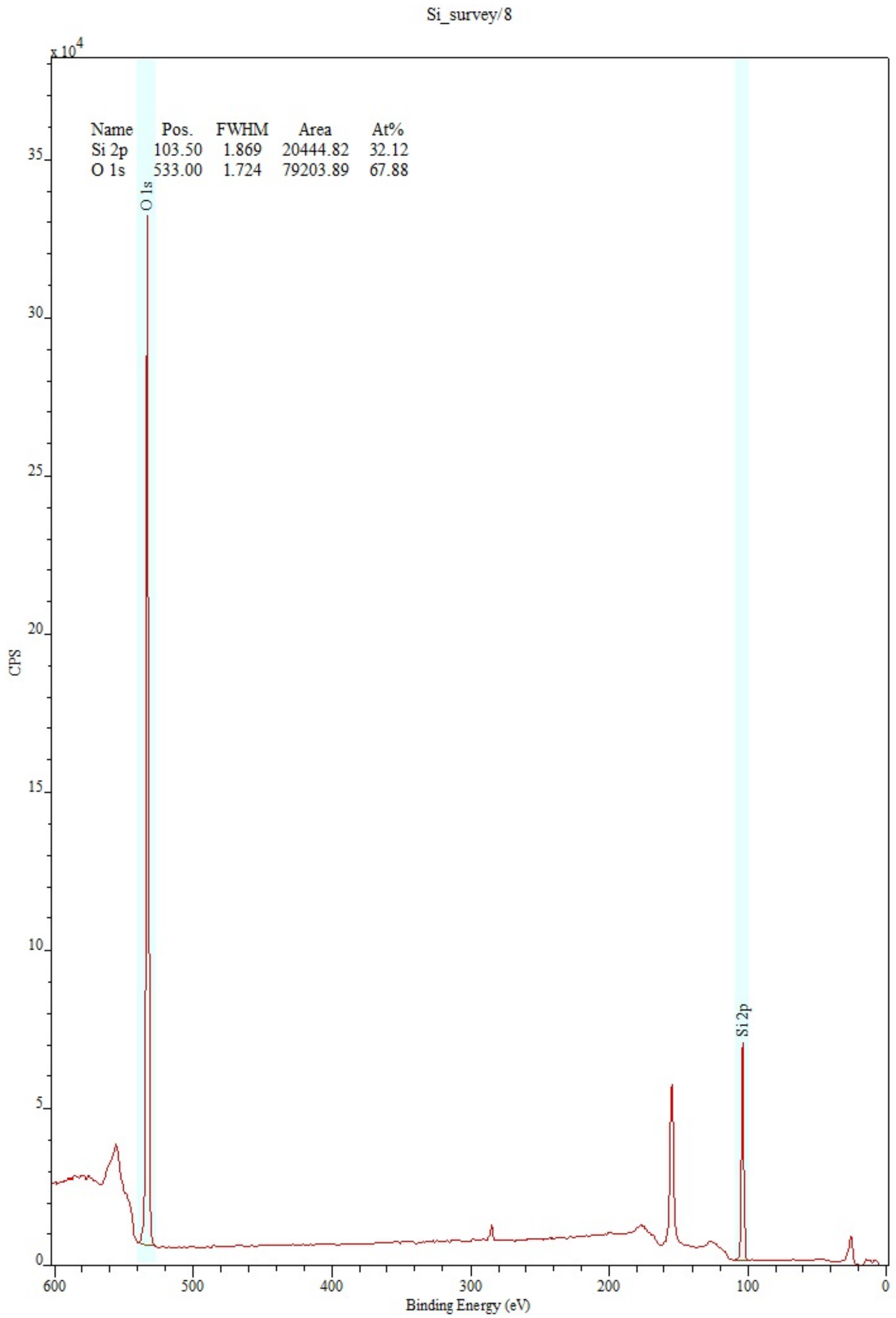

Supplementary Figure 24. XPS of silicon wafer survey.

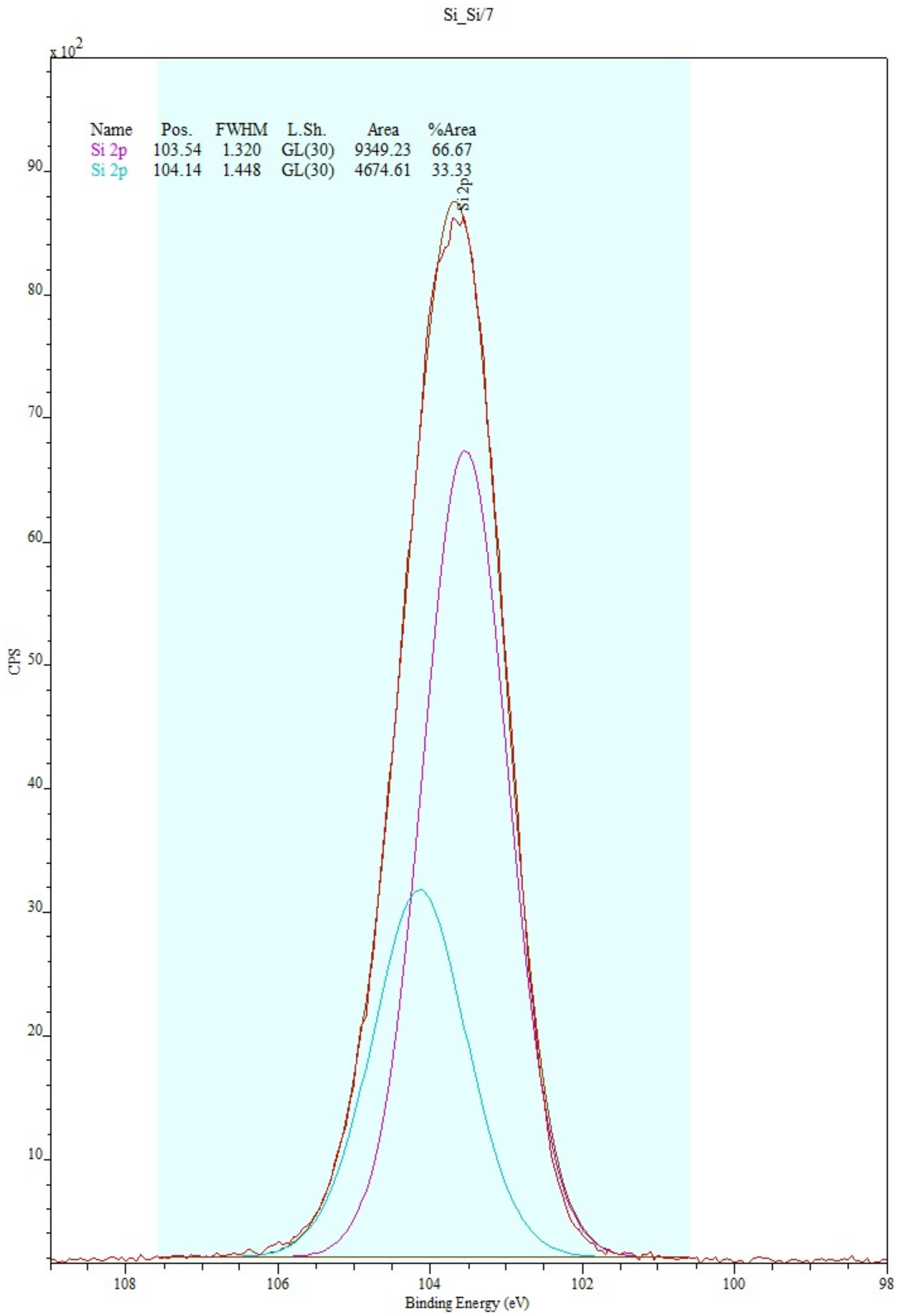

Supplementary Figure 25. XPS of silicon wafer, high resolution Si 2p.

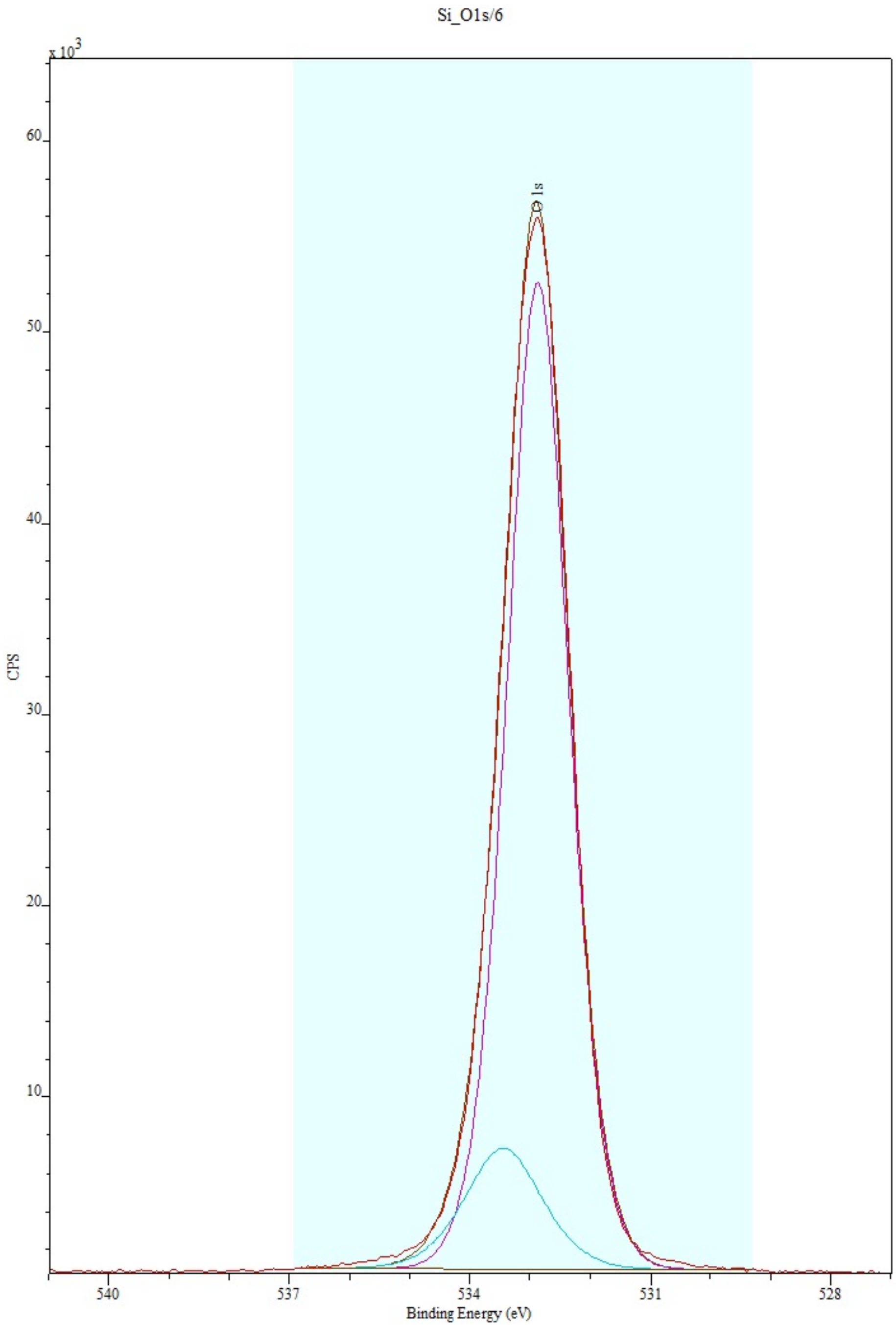

Supplementary Figure 26. XPS of silicon wafer, high resolution O 1s.

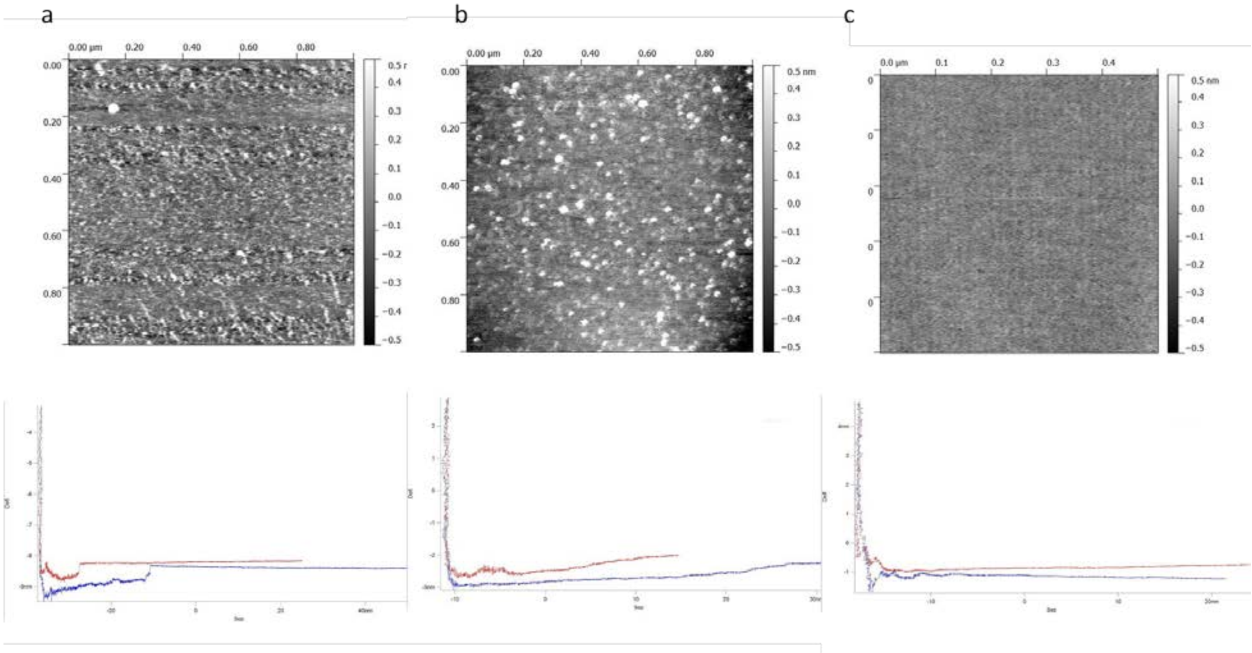

Supplementary Figure 27. AFM images of Z-Cat-C10 at different concentration: (a) 0.001 mM (below its CAC), (b) 0.05 mM (above CAC) and (c) 5 mM.

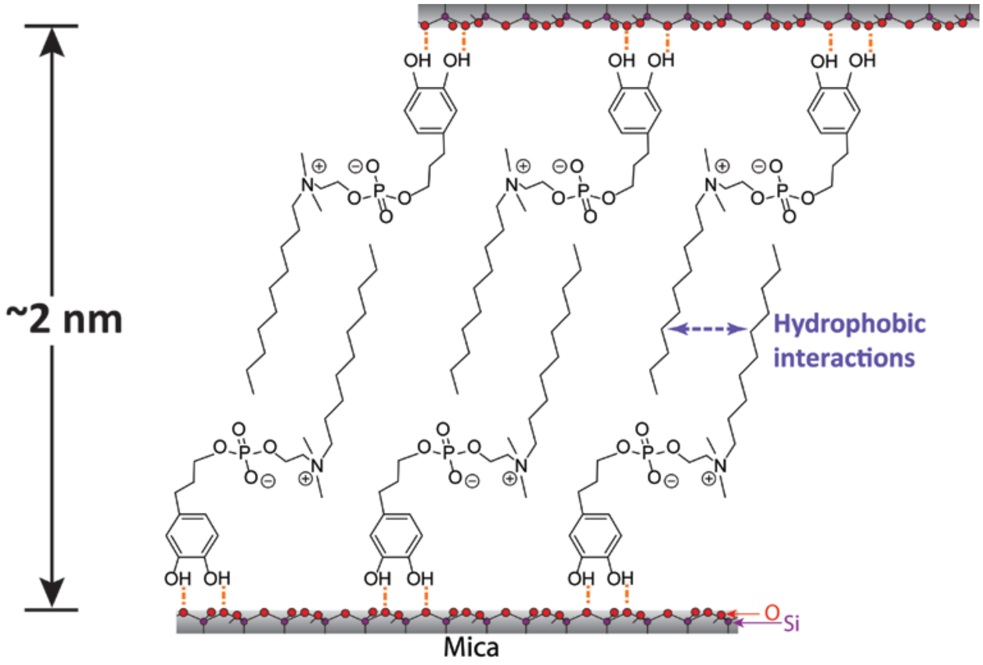

Supplementary Figure 28. Chemical configuration of Z-Cat-C10 on mica at low

concentrations.

Supplementary Notes

Supplementary Note 1

Although in general the unoptimized yields were satisfactory for the purposes of this work, if yield is an important parameter, the following can occasionally assist in improving the overall efficiency. For relatively volatile amines such as n-$C_4H_9NMe_2$, the yield of the reaction was observed to depend on the headspace in the flask during reflux. As the reaction is performed in a sealed flask under vacuum, greater head space in the flask presumably lead to condensation of the amine above the level of solvent and gave decreased yield. Furthermore a high quality schlenk valve is essential, as the vacuum manifold is active during the reaction even though the schlenk valve is closed, and a faulty valve will lead to evaporation of solvent and amine over the course of the reaction. When volatile amines are used as nucleophiles in this reaction the following steps must be taken to ensure adequate yield. 1. The use of additional MeCN as solvent to reduce headspace. 2. Increasing amine equivalents from 2 to 4. 3. Applying vacuum to the flask very briefly before re-sealing to avoid evaporation of amine. 4. Increasing reaction time from 2 to 4 days.

Supplementary Note 2

Although Menger's procedure documents the use of washing, and recrystallization to purify these compounds[1], in our hands recrystallization under the reported conditions led to only modest increases in purity, gave diminished yields and was highly dependent on the molecule being purified. As the last step of the synthesis (catalytic hydrogenolysis) was chosen to avoid the necessity of additional purification and introduction of additional impurities, and considering

that catechols in their unprotected state are highly polar, and prone to oxidation/polymerization, an additional purification step following catechol-de-protection was deemed undesirable and considerably more difficult. Key to the success of this work was ensuring a high level of purity *prior to de-protection of the catechols* and thus it is recommended that purification of benzylated catechol intermediates be performed with C2-bonded reverse phase silica gel.

Supplementary Note 3

"Reverse phase" C2 silica is technically a misnomer in this case as compounds of low polarity displayed low retention times eluting first, and compounds of high polarity displayed high retention times, eluting last. Gradient elution was performed starting with 0%MeOH/100%CH_2Cl_2 and the % of MeOH was increased over 10-25CV's to 35%MeOH/65%CH_2Cl_2. If cost of the reverse phase silica gel is of consideration, then C2 silica could be prepared readily by reacting the appropriate amount of ethyltrichlorosilane with standard grade silica gel (230 - 400 mesh) according to a previously described procedure.[1] However, C2 silica prepared by this route was considerably more polar than the commercial material obtained from Analtech, and required longer and larger, 0-100% MeOH/CH_2Cl_2 gradients to allow the desired product to elute. Both sources of C2 silica gel were used in this work and no substantial difference in the purity was observed with material purified with either source of C2 silica. To further reduce cost, the C2 silica from either source could be reused several times after use by flushing with 10-20 volumes of MeOH and storing the sealed columns wet with MeOH in a refrigerator. Before reuse, the columns were then flushed with 5-10 volumes of CH_2Cl_2 prior to loading crude compound. Without exception, in all cases the desired product was observed to elute last on the column, although on occasion the first few fractions of

those that contained desired material also contained unidentified yellow-colored impurities, and these fractions were either discarded or separated, concentrated, and repurified according to the procedure.

Supplementary Note 4

As the high polarity of the products precluded the use of TLC or GC to monitor the progress of the reaction, reaction progress was monitored at 24h intervals by removal of a 0.5-1.0 ml aliquot of the reaction mixture, which was worked up by filtration according to the procedure and analyzed by NMR to determine completion of the reaction. In general, most reactions were incomplete after one day, and showed full conversion after two full days, although on occasion, up to 4 days were necessary for certain substrates.

Supplementary Note 5

This procedure was devised on the basis of the expected high polarity of the products, which would preclude removal of Pd/C, by the usual filtration over celite, silica, or alumina, and thus a relatively inert and nonpolar PTFE filter was chosen to remove the Pd/C. The choice of the benzyl protecting group for catechols in this context is particularly noteworthy, as the only byproduct is toluene which can be removed by simple evaporation. Use of an acetonide or silicon based protecting group for the catechol, and subsequent removal with acid or fluoride respectively, was deliberately avoided, as they would introduce other organic, or highly polar water-soluble impurities, which would be difficult to remove from the desired product with conventional techniques. However this procedure gave variable (39-93%) isolated yields, presumably due to adsorption of the products onto the charcoal surface, and no attempt was made to optimize yields, although on occasion, additional washing of the reaction flask and

PTFE filter with degassed MeOH was performed to assist in product recovery.

Supplementary Note 6

Although azeotropic removal with pentanes, and gentle heating under high vacuum were successful at removing the majority of trace solvents from the pure coacervates, NMR spectra invariably contained some slight traces of solvents owing to the high propensity of the product molecules to self aggregate, trapping some residual solvents in the material.

Supplementary Discussion

For the macro-scale test, Z-Cat-C10 was dissolved at 0.1 mg ml^{-1} concentration in DI water (clear sample), then Z-Cat-C10 coacervate (phase-separated liquid) was injected into artificial sea water, periodate (Sigma Aldrich) solution (1M), DI for CV (**Supplementary Figure 2**) and UV-Vis (**Supplementary Figure 3**). In CV for the sample in DI water, its oxidation peak at 530 mV and a reduction peak at 316 mV appeared indicating catechol functionality. Moreover, an absorbance peak at 280 nm in the UV-Vis spectrum supported the presence of the catecholic structure. In artificial sea water (pink sample), alkaline pH and saturated oxygen conditions are an appropriate environment for catechol oxidation.

AFM images of Z-Cat-C10 at different concentrations: 0.001 mM (below its CAC), 0.05 mM (above CAC) and 5 mM, are shown in **Supplementary Figure 27**. The 5 mM concentration shows molecularly smooth surface and its chemical configuration is suggested in **Figure 4a**. The cartoon of its chemical configuration at low concentrations is suggested in **Supplementary Figure 28**.

Coacervates formed from 5 mM Z-Cat-C10 effectively coat mica surfaces as atomically smooth bilayers (**Figure 4c, d**). The catecholic functionalities interact with mica by H-bonding

and, between two bilayers, by intercatecholic H-bonding and aryl coupling following oxidative crosslinking (**Figure 4a-d**). In the absence of coacervation, at 0.001 mM Z-Cat-C10, which is below the CAC at 0.05 mM solution, Z-Cat-C10 forms monolayers that exhibit only weak adhesion and no cross-linking (**Supplementary Figure 27 and 28**).

QCM- Adsorption of the molecules on different surfaces such as silica, SiO_2, (**Supplementary Figure 5**) and titania, TiO_2, (**Supplementary Figure 6**) was also explored by the quartz crystal microbalance (QCM) and confocal fluorescence microscopy after rhodamine staining (**Supplementary Figure 7**). Quartz crystal microbalance with dissipation (QCM-D): A "static cell" (often called "Open Model") QCM-D (Q-Sense, Biolin Scientific) was employed to qualitatively show the adsorption of the zwitterionic molecules onto mineral and metal oxide surfaces. We deposited 50 µl of 5 mM zwitterionic solution onto TiO_2 and SiO_2 surface, respectively, in DI H_2O (100 µl) using a static cell via two-step adsorption. In this two-step adsorption, all the zwitterionic molecules adsorb onto both TiO_2 and SiO_2 (using quartz sensors with the corresponding top-layer. See Figures S2-1 and S2-2). Frequency and dissipation change upon addition of 5 mM solution of each zwitterionic molecule to TiO_2 and SiO_2 surface, respectively (See Figures S2-1 and S2-2). The results are in contrast to all the studied mussel foot proteins, which adsorb onto TiO_2 but not to SiO_2 according to previous QCM-D experiments (less than 10 ng cm^{-2}). The results emphasize qualitatively that all the zwitterionic molecules adsorb onto both TiO_2 and SiO_2, which is in agreement with the SFA and AFM results. However, a quantitative discussion regarding the adsorbed mass (frequency shift) and the viscoelastic properties (dissipation) of the adsorbed "soft" layers is challenging because the mass of the zwitterionic molecules cannot be separated from the mass of adsorbed water.

Z-Ben-C8 in the SFA- The catechol-containing homologs behaved differently from the

noncatecholic control (Z-Ben-C8). Z-Ben-C8 shows regular bilayer repulsion and jump-in patterns of a general surfactant when two surfaces were approached (see approach and separation patterns in **Supplementary Figure 8**), whereas catechol-containing molecules did not show a significant repulsion or no repulsion at all (see approach and separation patterns of the molecules in **Figure 3b**). Z-Ben-C8 had a CAC similar to that of Z-Cat-Cat (and W_c= 8.1±1.3mJ m^{-2}); however, the latter never formed more than a monolayer on mica.

SFA measurements were performed on molecules deposited at 0.5 mM concentration in DI water to test the effect of concentration on the interfacial cohesive forces between the thin films. Z-Ben-C8 (no catechol) solution (below its CAC) formed bilayer (2 nm) whereas the catechol-carrying small molecules formed monolayer (1 nm) (**Supplementary Figure 9**). The representative force *vs.* distance plots between mica surfaces of 0.5 mM aqueous despersions of Z-Cat-C10 (red points), -C8 (blue points), -C6 (pink points), -C4 (black points), and -Cat-Cat (green points), respectively (**Supplementary Figure 10**). The cohesive interaction energy, W_c, of all molecules did not change for contact times, t_c from 2min to 12h. Filled and open circles represent the forces measured during approach and separation of the surfaces respectively. The interaction energy of Z-Cat-C8 (~22 mJ m^{-2}) was very high at 0.5 mM concentration (**Supplementary Figure 11**).

The hard-wall thickness (approximately, the sum of the hydrodynamic diameters of the films on the upper and lower mica surfaces in SFA) of each homolog was also measured as the limiting distance between the mica surfaces during the approach run in the SFA. SFA experiments were performed for Z-Cat-C8 and Z-Cat-C10 at C = 0.001 and 0.005 mM, respectively, to test the effect of deposition concentrations below the critical aggregation concentration (CAC) on the measured interaction (adhesive and cohesive) forces. It should be

noted that in SFA measurements, W_{ad} or $W_c \rightarrow -E_{ad}$ or $-E_c$ respectively, where E is the energy of interaction between the surfaces. The reason for following this convention is because the measured adhesive or cohesive forces between the surfaces are attractive. We refer adhesion as the interaction between two asymmetric surfaces and cohesion as the interaction between two symmetric surfaces.

Atomic Force Microscopy (AFM) scans of a mica surface adsorbed with the small molecules from a solution in DI water of varying concentrations (5 mM). Z-Cat-C10 forms a defect free atomically smooth bilayer on mica. On the other hand, Z-Cat-C4, Z-Cat-C8, Z-Cat-10 and Z-Cat-Cat form small aggregates on the surface, and Z-Ben-C8 formed thick multilayer.

AFM force runs were measured with and without Z-Cat-C10 on silicon wafer and copper plate, respectively, vs. silicon dioxide probe. *Underwater* force run was conducted for the surface prepared subsequently after spreading the 5 mM Z-Cat-C10, waiting for 2min, rinsing thoroughly with degassed DI water thoroughly for 3 times. Dry force run was performed after ~5min dry of the surface prepared as for the *underwater* force run. Because adhesive force increased significantly after the glue layer dried for ~5min using a gas duster gently, the ϕ=10 μm probe stuck to $CuO_2/Cu(OH)_2$ surface, and the both ϕ=10 and 5 μm probe stuck to SiO_2 surface. Cantilever spring constant (K) for ϕ=10 μm silica tip on silicon wafer and copper plate with Z-Cat-C10, was 0.1 N m^{-1}.

Adhesion force (F_a) measured with ϕ=10 μm probe was ~2 nN at SiO_2 and $CuO_2/Cu(OH)_2$ interface and ~167 nN at SiO_2 and SiO_2 interface, respectively. The *underwater* adhesive force was measured with ϕ= 10 μm probe at $CuO_2/Cu(OH)_2$ and SiO_2 interface; F_a ~ 2 nN, and 1 μm probe at SiO_2 and SiO_2 interface; F_a ~ 170 nN, respectively. Dry adhesive force

increased significantly, thus measured with ϕ= 5 µm probe at $CuO_2/Cu(OH)_2$ and SiO_2 interface; F_a ~260 nN, and 1 µm probe at SiO_2 and SiO_2 interface; F_a ~150 nN, respectively.

The measured adhesion, F_a, force (minimum of the potential well of the F *vs*. D curves obtained from AFM measurements) is related to the adhesion energy per unit area by $W_a = F_a/2\pi R$ for rigid surfaces with weak adhesive interactions, and by $W_a = F_a/1.5\pi R$ (used in this study) for soft deformable surfaces with strong adhesion or cohesion[4]. Since copper surface (RMS ~2 nm) is rougher than silicon wafer (RMS <1 nm), the W_a on copper surface was comparatively weaker than the W_a of silicon wafer. Hydroxide/oxide on copper surface also weakens the adhesion to copper plate compared to just oxide on silicon wafer (see **Supplementary Figure 21-26**) This strong and defectless nano-glue layer hold particular promise for electronic devices to obviate the interfacial failure causes fatal multifunction.

Adhesion test- In the context of adhesion, adhesion energies and adhesion forces (or strengths) are very different; the first is unchangeable for a given system, whereas the latter can vary greatly just by slightly changing test parameters. The two are related by $W_{ad} = \int \vec{F}_{ad} d\vec{x}$, where W_{ad} is the adhesion energy, F_{ad} is the force, and x is the displacement of the two surfaces as they are separated. Note that F_{ad} and x are vectors, i.e. the angle at which the two surfaces are separated also affects the force for separation[28]. As a result, different paths for surfaces to separate yield very different adhesion forces, F_{ad}, but involve the same net changes in energy, i.e., adhesion energies, W_{ad}. Therefore, adhesion forces, F_{ad}, measured in different configurations, e.g., the lap joint rupture (force), three point bending peel strength (force per width), and shear strength (force per area), and/or measured on different surfaces, e.g., type of materials and surface roughness (determining actual contact area), are not interchangeable nor comparable to one another. For example, F_{ad} of peeling can be orders of magnitude less than F_{ad} of planner

separation, whereas W_{ad} in both cases is same[28]. Therefore, rather than comparing F_{ad} measured and reported in different configurations and/or on different surfaces, W_{ad} reported in this study was compared to previously published W_{ad} using the similar techniques, i.e., SFA and AFM, based on Johnson-Kendall-Roberts (JKR) theory between atomically smooth mica surfaces or between silica surfaces with RMS roughness <1 nm.

The lap joint bending peel test showed that Z-Cat-C10 prevented the rupture of the bonding between the steel plates for a load up to 0.3 kg *underwater* and 1.1 kg under *dry* ambient conditions when the joint prepared in artificial sea water and aqueous periodate solution, respectively. As a control, the joint made from 3M double sided scotch® tape under *dry* ambient conditions held only up to 0.3 kg under *dry* ambient conditions.

Supplementary Methods

Synthesis of the molecules

All synthetic manipulations were carried out under an atmosphere of Argon unless otherwise noted, and no attempt was made to optimize reaction yields. TLC plates (UV 254 indicator, glass backed, thickness 200 mm) and silica gel (standard grade, 230 – 400 mesh) were purchased from Merck. Bonded C2 reverse phase silica was prepared as previously described[2] or purchased from Analtech (Catalog number 08010). Normal phase flash chromatography was performed manually in glass columns. Reverse phase flash chromatography was performed with bonded C2 reverse phase silica hand packed in plastic columns and performed on a Biotage SP4 chromatography system. Diethyl ether, THF, ethyl acetate, and hexanes were purchased from Fisher Scientific. Diethyl ether and THF was taken from Innovative Technologies Solvent Purification System (SPS) and used immediately. Dimethylformamide (DMF) and Acetonitrile

(MeCN) were purchased pre-dried from Spectrum and stored over activated 4 Å or 3 Å molecular sieves respectively after opening. Et_3N was distilled and stored on activated 4 Å molecular sieves under argon. NMR solvents were purchased from Cambridge Isotopes Laboratories. NMR spectra were recorded at 23 °C on Varian Unity INOVA (500 and/or 600 MHz) spectrometers. NMR spectra were processed using the MestreNova software package and processed with automatic phase correction, and automatic baseline correction using Bernstein Polynomial fitting. Reported chemical shifts are referenced to residual solvent peaks[3]. Reported chemical shifts for multiplets are reported corresponding to the most downfield peak of the multiplet, where s = singlet, d = doublet, t = triplet, q = quartet, p = pentet, sex = sextet, sept = septet, m = multiplet, br = broad. IR spectra were acquired on a FTIR Perkin Elmer Spectrum Two: UATR Two spectrometer using 1 cm^{-1} resolution. High resolution mass analyses were obtained using a 5975C Mass Selective Detector, coupled with a 7890A Gas Chromatograph (Agilent Technologies) as capillary column a HP-5MS cross-linked 5% phenylmethyl-polysiloxanediphenyl column (30 m x 0.250 mm, 0.25 micron, Agilent Technologies) was employed. Helium was used as carrier gas at a constant flow of 1 ml min^{-1}.

Synthesis of Small-Molecule Zwitterionic Adhesieves:

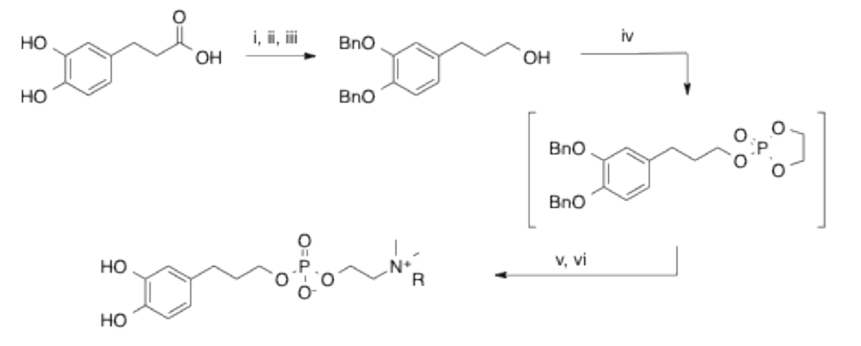

i) BnBr, DMF, K_2CO_3, 80˚C, 24h ii) NaOH, MeOH 100 ˚C 4h iii) $LiAlH_4$, THF, overnight iv) Ethylene chlorophosphate, Et_3N, Et_2O, 0˚, 4h v) $RNMe_2$, MeCN, 80˚C 2 days vi) H_2 (1atm), Pd/C, CH_2Cl_2/MeOH 2-4 days

Synthesis of starting materials

Benzyl 3-(3,4-bis(benzyloxy)phenyl)propanoate (2)

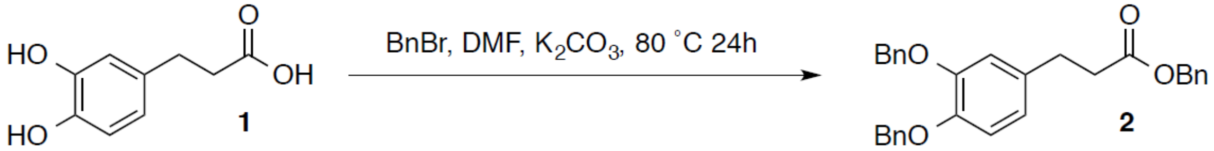

Benzyl 3-(3,4-bis(benzyloxy)phenyl)propanoate **2** was synthesized from 3-(3,4-dihydroxyphenyl)propanoic acid **1**, purchased from Alfa Aesar, according to a previously described procedure with slight modifications[4]. As the carboxylic acid contains potentially air sensitive catechol moieties, after opening, the bottle of carboxylic acid was purged with argon, and the cap tightly wrapped with parafilm until subsequent use. While the previously published procedure was observed to work well on scales < 5 grams, adequate stirring became problematic on the scales required for this work, and it is recommended that the procedure be performed with the largest possible football shaped stir bar that can fit into the flask. If stirring is observed to cease during the procedure due to caking of the base, one septa can be briefly removed while under positive Argon flow, and the solidified mass of K_2CO_3 at the bottom of the flask broken up gently with a dry metal spatula until stirring resumes, whereupon a fresh septa is added to the flask and the vessel stirred until completion on the reaction.

A flame dried 500 ml 3-necked round bottom flask was fitted with rubber septa and a large football shaped stir-bar and allowed to cool to ambient temperature under positive argon flow. Subsequently, 20 grams (1 equiv., 109.8 mmol) of 3-(3,4-dihydroxyphenyl)propanoic acid was added, followed by 200 ml of anhydrous DMF with stirring. Once dissolved, 90.9 grams of

anhydrous K_2CO_3 (6 equiv., 658.7 mmol) was added with stirring. Then, 58.678 ml of fresh benzyl bromide (4.5 equiv., 494 mmol) was added via syringe. The solution was placed in an oil bath set to 80˚C and stirred for 1 day at this temperature. After this time, no further reaction was observed by TLC, which also indicated the reaction was incomplete, and contained in addition to the desired product, a mixture of mono- and di-benzylated products. The reaction vessel was allowed to cool to room temperature. The reaction mixture was then poured through a large fritted glass funnel into a 2 L round bottom flask to remove solids, and the reaction vessel was rinsed 3x300 ml EtOAc through the frit. The solvent was then removed under reduced pressure with a rotary evaporator. To assist subsequent extraction, residual DMF was removed by 4 cycles of evaporation with toluene (500 ml). The crude residue was then redissolved in 1.5 L of Et_2O and washed 5x100 ml ice cold water, 1x500 ml Brine, dried over Na_2SO_4, and concentrated under reduced pressure. The crude residue was then dry-loaded onto silica gel and purified by flash chromatography gradient elution 10-40% Et_2O/hexanes in a large (18 inch tall) glass column. Fractions containing the only desired product were identified by TLC at R_f = 0.31 (20:80 Et_2O:Hexanes, Stain = UV/Seebach's stain), pooled, and concentrated under reduced pressure to yield 17.42 grams of desired product. Fractions containing the two regioisomeric dibenzylated products, in which the carboxylic acid and either the 3- or 4- hydroxyl was benzylated, were identified by TLC at R_f = 0.15 and 0.19 (20:80 Et_2O:Hexanes Stain = UV/Seebach's stain), pooled, concentrated under reduced pressure, and then resubjected to the reaction conditions to give an additional 20.56 grams of product, bringing the total amount of product to 37.98 grams in 76% isolated yield. The material was quickly checked for purity by ^{1}H-NMR and then carried on immediately to the next step. If the yield of the initial reaction is not deemed objectionable, after removal of solvent, rather than collecting partially benzylated

material and resubjecting it to the reaction conditions, the product can be more rapidly purified by 2 successive filtrations over a 6-8 inch tall pad of basic Al_2O_3 (Acros, 50-200µm) eluting with 20% Et_2O/Hexanes.

^{1}H NMR (500MHz, $CDCl_3$) δ (ppm): 7.45-7.41 (m, 4H) 7.38-7.27 (m, 11H), 6.85 (d, *J* = 8.5 Hz, 1H), 6.80 (d, *J* = 2 Hz, 1H), 6.70 (dd, *J* = 2, 8.5 Hz, 1H) 5.12 (s, 2H), 5.10 (s, 2H), 5.09 (s, 2H), 2.88 (t, *J* = 7.5 Hz, 2H), 2.63 (t, *J* = 7.5 Hz, 2H)

3-(3,4-bis(benzyloxy)phenyl)propanoic acid (3)

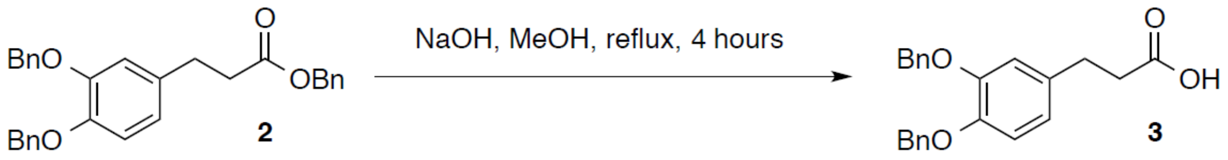

3-(3,4-bis(benzyloxy)phenyl)propanoic acid **3** was synthesized in 88% isolated yield by saponification of **2** as previously described. Spectral data matches that of previously reported[4].

^{1}H NMR (500MHz, $CDCl_3$) δ (ppm): 9.19 (s, 1H), 7.46-7.41 (m, 4H), 7.38-7.33 (m, 4H), 7.32-7.27 (m, 2H), 6.87 (d, *J* = 8.5 Hz, 1H), 6.80 (d, *J* = 2 Hz, 1H), 6.73 (dd, *J* = 2, 8 Hz, 1H), 5.14, (s, 2H), 5.13 (s, 2H), 2.87 (t, *J* = 7.5 Hz, 2H), 2.62 (t, *J* = 7.5 Hz)

3-(3,4-bis(benzyloxy)phenyl)propan-1-ol (4)

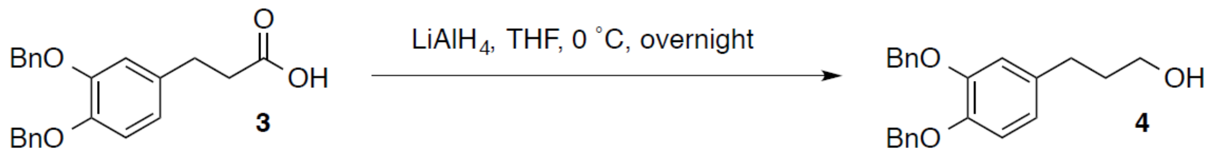

3-(3,4-bis(benzyloxy)phenyl)propan-1-ol **4** was synthesized from **3** in 89% isolated yield by

reduction with $LiAlH_4$. 7.24 grams of acid **3** (20 mmol, 1 equiv.) were dissolved in 100 ml of anhydrous THF and cooled to 0 ˚C in an ice bath. 3.04 grams of $LiAlH_4$ (80 mmol, 4 equiv.) was then added carefully in 4 portions. The reaction was left to stir overnight under argon while warming to ambient temperature. The reaction was then quenched cautiously according to the Feiser workup, diluted with 100 ml of Et_2O and the aluminum solids were filtered off. The solution was then transferred to a separatory funnel, washed once with saturated $NaHCO_3$, dried over Na_2SO_4, filtered, and evaporated under reduced pressure to afford crude material. The crude material was subsequently purified on a pad of silica eluting with Et_2O. The compound was isolated as a clear viscous oil which gradually solidified over one week under high vacuum to a white wax. Over time, a slight pink coloration developed on the surface of the wax but this did not adversely purity as determined by ^1H-NMR or negatively affect subsequent steps. Attempts to prepare this compound directly from the reduction of the corresponding benzyl ester led to unsatisfactory levels of purity, as benzyl alcohol co eluted with product in flash chromatography, while bulb to bulb distillation was inefficient and took extended times to reach a satisfactory level of purity. Spectral data matches that of previously reported[5].

^1H NMR (600MHz, $CDCl_3$) δ (ppm): 7.47-7.42 (m, 4H), 7.40-7.33 (m, 4H), 7.32- 7.28 (m, 2H), 6.87 (d, *J* = 7.8 Hz, 1H), 6.79 (d, *J* = 1.8 Hz, 1H), 6.71 (dd, *J* = 1.5, 8.1 Hz, 1H), 5.15 (s, 2H), 5.13 (s, 2H), 3.62 (q, *J* = 6 Hz, 2H), 2.62 (t, *J* = 7.5 Hz, 2H), 1.84 (p, *J* = 7.2 Hz, 2H), 1.18 (t, *J* = 5.7 Hz, 1H).

Procedure for preparation of dimethylamide (5)

Dimethylamide **5** was conveniently prepared with 1-1' carbonyldiimidazole as peptide coupling

reagent. A flame dried flask was fitted with a PTFE coated stir bar, rubber septa, and let cool to ambient temperature under positive argon flow. 1 equiv. of the corresponding carboxylic acid, 4 equiv. of anhydrous Et_3N, and anhydrous CH_2Cl_2 [0.5M] were added to the flask successively. The flask was cooled to 0 ˚C in an ice bath and stirred briefly, whereupon 1-1' carbonyldiimidazole (1.1 equiv.) was added portionwise, (gas evolution) the cooling bath was then removed, and the solution was stirred for an additional 30 minutes while warming to ambient temperature. Finally Dimethylamine as the hydrochloride salt, (2 equiv.), was added in one portion and the solution was stirred until TLC indicated completion. Upon completion the contents of the reaction vessel were transferred to a separatory funnel, diluted with CH_2Cl_2, and the organic layer was washed 2 x 1N HCl, 2 x sat. $NaHCO_3$, and dried over Na_2SO_4. The organic layer was filtered, evaporated under reduced pressure, and the crude residue was filtered once over a pad of basic Al_2O_3 eluting with EtOAc, evaporated again, and purified by flash chromatography gradient elution with 50-100% EtOAc/Hexanes. Dimethylamide **5** was obtained in high purity as determined by TLC, and not fully characterized at this stage as it was carried immediately on to the next step. Yield was not optimized.

3-(3,4-bis(benzyloxy)phenyl)-*N*,*N*-dimethylpropanamide (5)

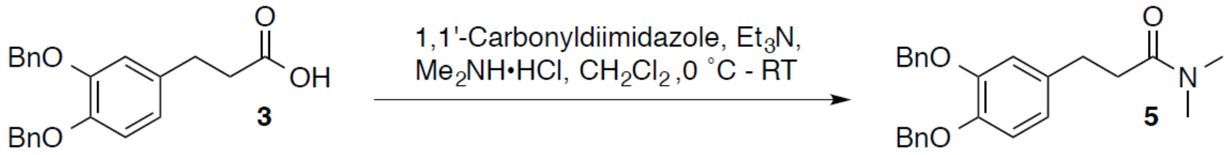

Prepared according to the procedure given above in 76% isolated yield, and carried immediately on to the next step.

^{1}H NMR (600MHz, $CDCl_3$) δ (ppm): 7.46-7.44 (t, *J* = 7.2 Hz, 4H), 7.37-7.34 (dd, *J* = 3, 7.2 Hz,

4H), 7.32-7.29 (m, 2H), 6.87 (d, *J* = 8.4 Hz, 1H), 6.83 (d, *J* = 1.8 Hz, 1H), 6.74 (dd, *J* = 1.8, 8.4 Hz, 1H), 5.15 (s, 2H), 5.14 (s, 2H), 2.93 (s, 3H), 2.88 (m, 5H), 2.54 (t, *J* = 8.4 Hz, 2H)

3-(3,4-bis(benzyloxy)phenyl)-*N*,*N*-dimethylpropan-1-amine (6)

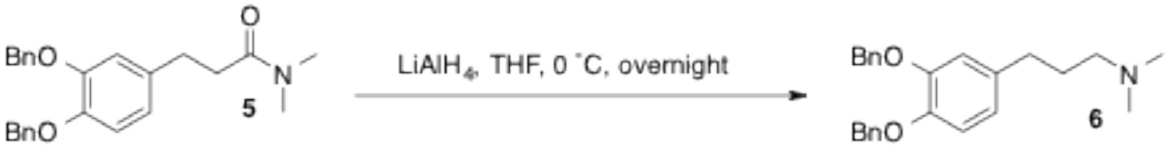

Prepared according to the general procedure in 87% isolated yield

^{1}H NMR (600MHz, $CDCl_3$) δ (ppm): 5.15 (s, 2H), 5.13 (s, 2H), 2.54 (t, *J* = 7.8 Hz, 2H), 2.25 (t, *J* = 7.2 Hz, 2H), 2.20 (s, 6H), 1.74 (p, *J* = 7.8 Hz, 2H)

^{13}C NMR (125MHz, $CDCl_3$) δ (ppm): 148.96, 147.67, 137.37, 137.20, 132.55, 128.46, 128.45, 127.82, 127.81, 127.40, 127.35, 121.29, 115.49, 115.42, 71.41, 71.27, 68.53, 62.04, 49.43, 31.50, 24.13

FTIR (cm^{-1}): 3088, 3058, 3031, 2939, 2857, 2814, 2763, 1605, 1588, 1509, 1454, 1423, 1379, 1261, 1221, 1158, 1134, 1067, 1016, 907, 847, 731, 694, 624, 605, 463

ESI-HRMS: Calculated for $C_{25}H_{30}NO_2^+$: 376.2271. Found: 376.2266 $(M+H)^+$

General procedure for the synthesis of benzyl-protected molecules by the Chabrier Reaction 7-13.

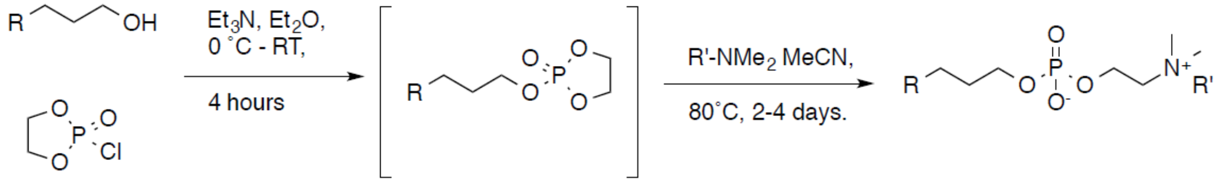

Benzyl-protected zwitterionic coacervates were prepared via the Chabrier-Reaction[6], according

to a previously described procedure[1], with certain modifications, and no attempt was made to optimize yields. Ethylene chlorophosphonate was purchased from Aesar, stored in a freezer, and used as received. In a typical procedure, a flame-dried flask was fitted with a PTFE coated stir bar, rubber septa, and cooled under positive argon flow. Freshly prepared alcohol was added to the flask followed by anhydrous Et_2O [0.4 M], 1.15 equiv. Et_3N, and stirred under argon in an ice bath. 1.15 equiv. ethylene chlorophosphonate was then added semi-dropwise via syringe whereupon precipitation of the amine hydrochloride salt was observed to begin, and the flask was stirred for 10 minutes at 0 ˚C. The ice bath was then removed and the flask was allowed to warm to ambient temperature with stirring for 4 hours. Hexanes equal to the volume of Et_2O in the flask, was then added to assist in precipitation of the amine hydrochloride salt, and the contents of the flask were filtered quickly over a pad of basic celite into a fresh round bottom flask. The contents of the reaction vessel was then rinsed once with hexanes, and once with Et_2O through the pad of basic celite, and volatiles were then removed under reduced pressure, and stored briefly in the round bottom flask under high vacuum while a second reaction vessel was prepared.

A schlenk-bomb type flask was fitted with a PTFE-coated stir bar, flame dried, fitted with two rubber septa, and allowed to cool to ambient temperature under positive argon flow. While the schlenk flask was cooling, the flask containing the phosphonate ester was back-filled with argon, removed from the vacuum manifold, fitted with a rubber septa, and an argon needle was inserted into the septum. The appropriate amount of anhydrous MeCN (2-4 ml per mmol alcohol) was then added to this flask via syringe, and swirled gently by hand until completely dissolved. At this point, the MeCN solution containing the phosphonate ester was transferred via syringe into the schlenk flask, and the round bottom was rinsed once with a minimal amount of

MeCN into the schlenk flask. 2-4 equiv. of the appropriate amine was then added to the schlenk flask, and the rubber septa was replaced with a schlenk valve coated with high-vacuum grease. The schlenk valve was closed, whereupon the second rubber septa containing an argon needle was replaced with a glass adaptor connected to the high vacuum manifold and placed under high vacuum. The schlenk valve was then cautiously opened and atmosphere was removed from the flask for 10 seconds to remove atmosphere from the flask, the schlenk valve was then closed tightly, and the flask was refluxed under vacuum with stirring for 2-4 days at 80 ˚C in an oil bath (see **Supplementary Note 1**).

When the indicated time had been reached the flask was removed from the oil bath and allowed to cool to ambient temperature. The flask was then backfilled with argon, removed from the vacuum manifold and the schlenk valve was removed. As the inside neck of the flask contained residual vacuum grease from the schlenk valve, to avoid contamination with this potential impurity, rather than pouring, the reaction mixture was transferred via syringe into a round bottom flask and the reaction vessel was washed twice with CH_2Cl_2 into the round bottom flask. Volatiles were removed under reduced pressure and traces of solvents were removed by several rounds of evaporation with pentanes to give the crude, protected coacervates. The residue was then dissolved in a minimum amount of CH_2Cl_2 and loaded on top of a plastic column packed with bonded C2 reverse phase silica (see **Supplementary Note 2**). The column containing the crude residue was then capped and purified on a Biotage SP4 column chromatography system with gradient elution from 0-35% MeOH/CH_2Cl_2 (see **Supplementary Note 3**) collecting the set of UV active fractions (254nm, 10mAu threshold) eluting last. Concentration of these fractions afforded pure benzyl protected coacervates which were characterized by HRMS, FTIR, ^{1}H-, and ^{13}C-NMR prior to de-protection.

Phospholane Intermediate

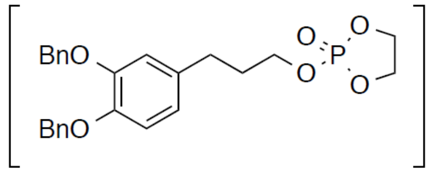

The cyclic phospholane intermediate in all cases was used immediately after preparation without further purification. However ^{1}H-NMR shifts of this crude material are included here for reference purposes and completeness of the supplementary information.

^{1}H NMR (500MHz, $CDCl_3$) δ (ppm): 7.46-7.42 (m, 4H) 7.38-7.33 (m, 4H), 7.32-7.28 (m, 2H), 6.87 (d, *J* = 6.5 Hz, 1H), 6.79 (d, *J* = 1.5 Hz, 1H), 6.70 (dd, *J* = 1.5, 6.5 Hz, 1H), 5.15 (s, 2H), 5.13 (s, 2H), 4.47 (m, 2H), 4.35 (m, 2H), 4.13 (dt, *J* = 2, 5 Hz, 2H), 2.63 (t, *J* = 6.5 Hz, 2H), 1.97 (p, *J* = 6 Hz, 2H)

Z-Cat-C_4-Bn (7)

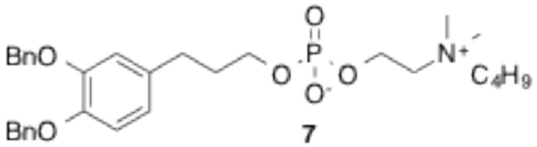

7

91% isolated yield.

^{1}H NMR (600MHz, $CDCl_3$) δ (ppm): 7.47-7.42 (m, 4H), 7.37-7.33 (m, 4H), 7.32-7.28 (m, 2H), 6.87-6.84 (m, 2H), 6.72 (dd, *J* = 7.8 Hz, 1H), 5.12 (s, 2H), 5.10 (s, 2H), 4.26 (m, 2H), 3.88 (q, *J* = 7.2 Hz, 2H), 3.71 (m, 2H), 3.44 (m, 2H), 3.26 (s, 6H), 2.62 (t, *J* = 9 Hz, 2H), 1.91 (p, *J* = 9Hz, 2H), 1.66 (m, 2H), 1.38 (sex, *J* = 9 Hz, 2H), 0.95 (t, *J* = 9 Hz, 3H)

^{13}C NMR (150MHz, $CDCl_3$) δ (ppm): 148.91, 147.24, 137.55, 137.44, 135.70, 128.49, 128.48, 127.84, 127.79, 127.55, 127.41, 121.44, 116.05, 115.46, 71.58, 65.44, 64.71, 64.04, 58.93, 51.67, 32.75, 32.66, 31.70, 24.68, 19.67, 13.74

FTIR (cm^{-1}): 3031, 2937, 2875, 1588, 1510, 1454, 1424, 1380, 1247, 1158, 1135, 1087, 1064, 1042, 982, 937, 806, 731, 695, 623, 536, 486

HRMS: Calculated for $C_{31}H_{42}NaNO_6P^+$: 578.2642 Found: 578.2637 $(M+Na)^+$

Z-Cat-C$_6$-Bn (8)

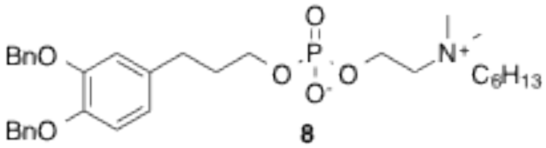

8

46% isolated yield.

^{1}H NMR (600MHz, $CDCl_3$) δ (ppm): 7.44-7.40 (m, 4H), 7.35-7.26 (m, 6H), 6.84-6.83 (m, 2H), 6.70-6.68 (m, 1H), 4.24 (m, 2H), 3.85 (q, *J* = 6 Hz, 2H), 3.69 (m, 2H), 3.39 (m, 2H), 3.24 (s, 6H), 2.60 (t, *J* = 7.8 Hz, 2H), 1.88 (p, *J* = 7.2 Hz, 2H), 1.63 (m, 2H), 1.35-1.22 (m, 8H), 0.87 (t, *J* = 7.2 Hz, 3H)

^{13}C NMR (150MHz, $CDCl_3$) δ (ppm): 148.97, 147.31, 137.61, 137.51, 135.77, 128.54, 128.53, 127.88, 127.84, 127.60, 127.46, 121.50, 116.14, 115.53, 71.69, 71.64, 65.86, 64.26, 58.92, 53.20, 51.77, 50.46, 32.78, 31.75, 26.03, 22.84, 22.50, 14.01

FTIR (cm^{-1}): 3039, 2955, 2931, 2259, 2200, 1588, 1510, 1454, 1425, 1379, 1250, 1136, 1088, 1046, 967, 906, 807, 722, 696, 640, 599, 538, 487

HRMS: Calculated for $C_{33}H_{46}NaNO_6P^+$: 606.2955 Found: 606.2949 (M+Na)

Z-Cat-C$_8$-Bn (9)

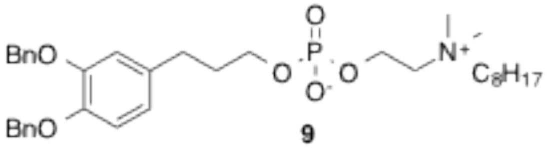

9

83% isolated yield.

^{1}H NMR (600MHz, $CDCl_3$) δ (ppm): 7.47-7.38 (m, 4H), 7.35-7.26 (m, 6H), 6.86-6.81 (m, 2H), 6.70 (dd, *J* = 1.8, 7.8 Hz, 1H), 5.10 (s, 2H), 5.09 (s, 2H), 4.25 (m, 2H), 3.86 (q, *J* = 6 Hz, 2H), 3.69 (m, 2H), 3.38 (m, 2H), 3.24 (s, 6H), 2.61 (t, *J* = 7.8 Hz, 2H), 1.89 (p, *J* = 7.2 Hz, 2H), 1.62 (m, 2H), 1.33-1.17 (m, 12H), 0.86 (t, *J* = 7.2 Hz, 3H)

^{13}C NMR (150MHz, $CDCl_3$) δ (ppm): 148.98, 147.32, 137.63, 137.53, 135.80, 128.55, 128.54, 127.88, 127.84, 127.60, 127.46, 121.51, 116.16, 115.53, 71.70, 71.65, 65.90, 64.84, 64.80, 58.91, 51.79, 32.78, 32.73, 31.76, 29.29, 29.16, 26.40, 22.91, 22.69, 14.18

FTIR (cm^{-1}): 3071, 3034, 2926, 2856,1651, 1589, 1511, 1455, 1425, 1379, 1221, 1159, 1136, 1081, 1039, 972, 733, 695, 539, 491

ESI-HRMS: Calculated for $C_{35}H_{50}NNaO_6P^+$: 634.3268 Found: 634.3243 $(M+Na)^+$

Z-Cat-C_{10}-Bn (10)

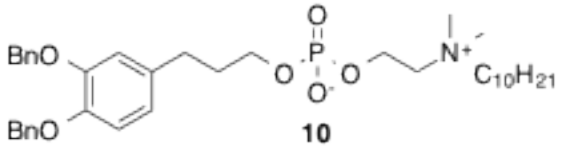

10

81% isolated yield.

^{1}H NMR (600MHz, $CDCl_3$) δ (ppm): 7.47-7.38 (m, 4H), 7.37-7.24 (m, 6H), 6.86-6.81 (m, 2H), 6.70 (dd, *J* = 1.8, 8.4 Hz, 1H), 5.10 (s, 2H), 5.08 (s, 2H), 4.25 (m, 2H), 3.86 (q, *J* = 6 Hz, 2H), 3.70 (m, 2H), 3.38 (m, 2H), 3.24 (s, 6H), 2.61 (t, *J* = 7.8 Hz, 2H), 1.88 (p, *J* = 7.2 Hz, 2H), 1.62, (m, 2H), 1.35-1.17 (m, 14H), 0.87 (t, *J* = 7.2 Hz, 3H)

^{13}C NMR (150MHz, $CDCl_3$) δ (ppm): 148.98, 147.32, 137.63, 137.53, 128.54, 128.53, 127.88, 127.83, 127.60, 127.45, 121.51, 116.15, 115.53, 71.69, 71.64, 65.88, 65.86, 64.85, 64.81, 58.98, 58.95, 51.77, 32.77, 32.71, 31.95, 31.75, 29.55, 29.51, 29.35, 22.92, 22.77, 14.18

FTIR (cm^{-1}): 3071, 3031, 2924, 2854, 1657, 1589, 1511, 1454, 1425, 1379, 1243, 1159, 1137, 1081, 1040, 1026, 967, 805, 789, 732, 695, 539, 489

ESI-HRMS: Calculated for $C_{37}H_{54}NNaO_6P^+$: 662.3581. Found: 662.3586 $(M+Na)^+$

Z-Cat-C_{12}-Bn (11)

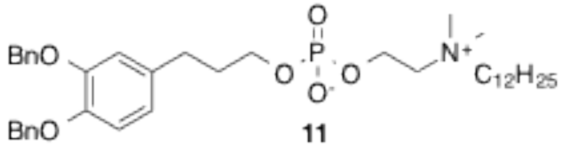

11

45% isolated yield.

^1H NMR (600MHz, $CDCl_3$) δ (ppm): 7.40-7.32 (m, 4H), 7.29-7.24 (m, 4H), 7.23-7.19 (m, 2H), 6.78-6.75 (m, 2H), 6.63 (dd, *J* = 1.2, 8.4 Hz, 1H) 5.04 (s, 2H), 5.02 (s, 2H), 4.18 (m, 2H), 3.84 (m, 2H), 3.68 (m, 2H), 3.31 (m, 2H), 3.17 (s, 6H), 2.53 (t, *J* = 7.8 Hz, 2H), 1.81 (p, *J* = 7.2 Hz, 2H), 1.61 (m, 2H), 1.29-1.06 (m, 16H), 0.81 (t, *J* = 7.2 Hz, 3H)

^{13}C NMR (150MHz, $CDCl_3$) δ (ppm): 148.98, 147.30, 137.61, 137.51, 135.76, 128.52, 128.51, 127.81, 127.58, 127.43, 121.47, 116.11, 115.52, 71.67, 71.61, 65.78, 64.81, 64.77, 64.25, 58.91, 51.74, 32.78, 32.73, 32.00, 31.75, 29.71, 29.62, 29.53, 29.43, 29.37, 26.41, 22.91, 22.78, 14.22

FTIR (cm^{-1}): 3071, 3028, 2923, 2853, 1589, 1512, 1455, 1425, 1378, 1227, 1137, 1084, 1040, 1026, 971, 721, 694, 492

ESI-HRMS: Calculated For $C_{39}H_{58}NNaO_6P^+$: 690.3894. Found: 690.3884 $(M+Na)^+$

Z-Cat-Cat-Bn (12)

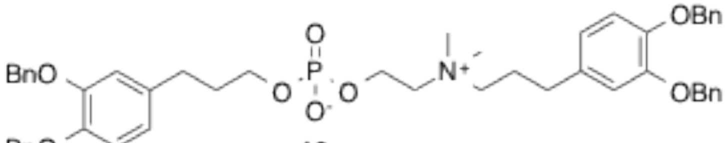

12

72% isolated yield. Samples of **12** were observed to degrade over time, and immediately after purification it was carried on to the next step.

^{1}H NMR (600MHz, $CDCl_3$) δ (ppm): 7.47-7.37 (m, 8H), 7.35-7.23 (m, 12H), 6.87-6.74 (m, 4H), 6.71-6.63 (m, 2H), 5.17-5.03 (m, 8H), 3.89 (s, 1H), 3.58 (m, 2H), 3.29 (m, 2H), 3.09-2.95 (m, 3H), 2.90-2.72 (m, 6H), 2.30-2.16 (m, 4H), 1.69-1.47 (m, 4H)

FTIR (cm^{-1}): 3031, 2941, 1588, 1510, 1454, 1425, 1380, 1259, 1218, 1159, 1136, 1078, 1011, 968, 848, 807, 733, 695, 466

HRMS: Calculated for $C_{50}H_{56}NNaO_8P^+$: 842.3636 Found: 852.3635 $(M+Na)^+$

Z-Ben-C_8 (13)

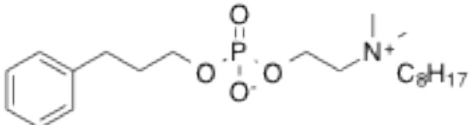

13

64% isolated yield.

^{1}H NMR (600MHz, $CDCl_3$) δ (ppm): 7.18-7.14 (m, 2H), 7.10-7.04 (m, 3H), 4.17 (m, 2H), 3.79 (q, *J* = 6.6 Hz, 2H), 3.38 (m, 2H), 3.21 (s, 6H), 2.60 (t, *J* = 7.8 Hz, 2H), 1.85 (p, *J* = 7.2 Hz, 2H), 1.65-1.52 (m, 2H), 1.29-1.06 (m, 10H), 0.79 (t, *J* = 6.3 Hz, 3H)

^{13}C NMR (150MHz, $CDCl_3$) δ (ppm): 141.83, 128.32, 128.23, 125.66, 65.37, 64.65, 63.97,

63.90, 58.77, 52.86, 52.52, 32.57, 32.53, 32.07, 31.57, 29.15, 28.99, 26.26, 22.75, 22.47, 13.97

FTIR (cm^{-1}): 3029, 2924, 2854, 1604, 1496, 1468, 1453, 1378, 1246, 1093, 1063, 1034, 979, 947, 905, 826, 795, 752, 743, 699, 594, 540, 498, 485.

ESI-HRMS: Calculated for $C_{21}H_{38}NNaO_4P^+$: 422.2431. Found: 422.2430 $(M+Na)^+$

General procedure for de-protection of catechols by hydrogenolysis 14-19

The oxidative stability of each of the zwitterionic coacervates containing unprotected catechols, either in the solid state, a solution in D_6-DMSO, or as a colloidal dispersion in water, was not known prior to undertaking this study and thus every effort was made to exclude atmospheric oxygen during all manipulations at all points after the catechols had been deprotected. Likewise, as purification of unprotected catechols was envisaged to be difficult and require application of purification techniques under inert atmosphere, every effort was made to increase the purity of the intermediates immediately preceding the de-protection step and the final products were all obtained in satisfactory purity as determined by FTIR, ^{1}H-, and ^{13}C-NMR Spectroscopy.

In a typical procedure, A Schlenk-bomb type flask was fitted with a PTFE coated stir bar, flame dried, fitted with two rubber septa and allowed to cool to ambient temperature under positive argon flow. One septa was briefly removed, 10-20 wt% of Pd/C (5%Pd, Type 87L, dry, Aesar) relative to mass of substrate was added to the flask and the septa was resealed. A small quantity of CH_2Cl_2 (4-8 ml) was added via syringe through the septa to rinse residual Pd on the sides of the flask to the bottom. A separate round bottom flask containing the desired amount of substrate was fitted with a rubber septa and argon needle, and vented briefly to purge air out. The vent needle was then removed, and the appropriate volume of a 1:1 v/v mixture of CH_2Cl_2/MeOH was added through the septa via syringe. The flask was then swirled by hand

until the benzyl-protected coacervate had dissolved, and this solution containing the substrate was transferred via syringe to the schlenk flask. The interior of the round bottom flask was then rinsed with a small quantity of MeOH (4-8 ml), and transferred via syringe to the schlenk flask. The first septa over the threaded part of the flask was quickly removed and replaced with a schlenk valve coated with vacuum grease. The schlenk valve was closed, whereupon the second rubber septa was replaced with a glass adaptor connected to the high vacuum manifold and placed under high vacuum, which placed the antechamber before the schlenk valve under vacuum, and the flask was stirred gently. The schlenk valve was then cautiously opened placing the contents of the flask under vacuum and the atmosphere was removed under vacuum for 2-3 minutes. Once this time had elapsed the schlenk valve was closed, the antechamber before the valve was backfilled with argon and the glass adaptor connecting the flask to the vacuum manifold was quickly replaced with a rubber septa. A hydrogen balloon (double ballooned) connected to a needle was placed through the septa and then a vent needle was placed though the septa to purge argon from the antechamber for 30 seconds whereupon it was subsequently removed. Then the schlenk valve was opened slowly to allow hydrogen into the reaction vessel. Stirring was continued for 2-4 days, with periodic replacement of the hydrogen balloon (fresh balloons were used with every replacement)(see **Supplementary Note 4**).

Once the indicated time had elapsed the schlenk valve was closed, and the remaining septa was replaced with a vacuum adaptor connected to a vacuum manifold and the antechamber before the schlenk valve was placed under vacuum. The schlenk valve was then cautiously opened placing the contents of the flask under vacuum and hydrogen gas was removed from the system in this manner for 5-10 minutes with stirring, whereupon there was concomitant bubbling and cooling of the flask due to slight solvent evaporation (Caution: opening the schlenk valve

too quickly in this step will lead to solvent “bumping” into the vacuum manifold). During this time a separate round bottom flask was flame dried, fitted with a rubber septa, tared, and allowed to cool to ambient temperature under positive argon flow. The schlenk flask was then backfilled with argon, and while under positive argon flow the schlenk valve was removed and quickly replaced with a rubber septa. A 30 ml, luer lock, PTFE coated syringe was fitted with a long metal needle, and the syringe was filled and purged with argon 3x, whereupon it was inserted through the septa of the reaction vessel. The Pd/C was then separated from the reaction mixture as follows: (see **Supplementary Note 5**)

With the outlet of the syringe facing down, 25 ml of the reaction mixture was pulled slowly up into the syringe, whereupon the needle was gently bent and the syringe was inverted so that the outlet of the syringe was now facing up. The needle was pulled above the level of solvent in the reaction mixture and a 5 ml blanket of argon pulled into the syringe. Then, very quickly, the needle was removed from the flask with the syringe still inverted, and the metal needle was removed from the luer lock and quickly replaced with an Acrodisc 0.45μm PTFE membrane filter fitted with a fresh 18 gauge needle at the outlet. The empty round bottom flask, still fitted with septa and argon needle, was then inverted so that the neck of the flask was facing downward, and the needle attached to the membrane filter and syringe was placed though the septa of the inverted flask. The whole apparatus was inverted once more, so that the outlet of the syringe was facing down and the neck of the flask was facing up, and the solution was gently forced through the filter into the flask, removing the Pd/C from the solution. If more than 25 ml of solution were present in the schlenk flask, then the procedure was repeated with fresh syringes, needles, and filters, until no more liquid remained in the flask. The septa was then quickly removed from round bottom flask containing product, and immediately placed on a

rotavap to remove volatiles. Several subsequent rounds of evaporation first with CH_2Cl_2, then with pentanes helped to remove trace solvents from the products, and the flask was immediately placed under high vacuum afforded pure de-protected coacervates, which were subject to further analysis and study (see **Supplementary Note 6**). Pure coacervates were both stored in round bottom flasks under high vacuum, or in vials under an argon atmosphere and tightly wrapped with several layers of parafilm, until further study. Unfortunately the final products were not sufficiently stable under conditions of EI- or ESI-MS for accurate mass determination. However they were all characterized by ^{1}H-NMR, ^{13}C-NMR, and IR spectroscopy, which confirmed that the anticipated products had been produced in satisfactory purity. The benzyl-protected coacervates were all sufficiently stable for ESI-HRMS (QTOF2 Tandem Mass Spectrometer) and were fully characterized including this descriptor prior to hydrogenolysis. Additionally, MALDI-MS (Operational settings on a model DE-VoyagerPerseptive Biosystems mass spectrometer were 25,000V (accelerating voltage), 93% (grid voltage), guide wire voltage (0.3%), 500 ns (delay time) and a relative laser power of 1000.), although not sensitive enough for an accurate mass determination, was performed successfully for Z-Cat-C8, which showed the anticipated molecular ion.

Z-Cat-C_4 (14)

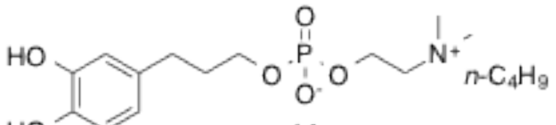

14

Reaction time = 2 days (48 hours) 88% isolated yield.

^{1}H NMR (500MHz, D_6-DMSO) δ (ppm): 9.67-8.74 (s, 1H), 9.67-8.74 (s, 1H) (overlapping),

6.76-6.58 (m, 2H), 6.46-6.37 (m, 1H), 4.06 (m, 2H), 3.68 (m, 2H), 3.51 (m, 2H), 3.35 (m, 2H), 3.07 (s, 6H), 2.44 (t, J = 7.5 Hz, 2H),1.77-1.58 (m, 4H), 1.32-1.20 (m, 2H), 0.92 (t, J = 7.5 Hz, 3H)

^{13}C NMR (125MHz, D_6-DMSO) δ (ppm): 145.17, 143.29, 132.31, 118.63, 115.81, 115.51, 63.95, 63.83, 63.02, 58.19, 58.15, 50.74, 30.91, 23.76, 19.17, 13.50

FTIR (cm^{-1}): 3029, 2959, 1599, 1513, 1468, 1382, 1286, 1202, 1079, 1059, 1035, 977, 813, 768, 733, 634, 588, 535, 492

Z-Cat-C_6 (15)

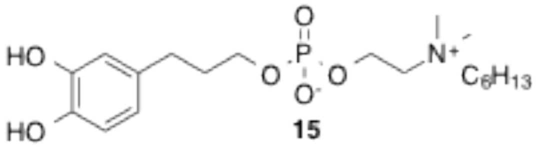

15

Reaction time = 2 days (48 hours) 74% isolated yield.

^{1}H NMR (600MHz, D_6-DMSO) δ (ppm): 9.79-8.80 (br, s, 1H), 9.79-8.80 (br, s, 1H) (overlapping), 6.69-6.58 (m, 2H), 6.44-6.33 (m, 1H), 4.04 (m, 2H), 3.69-3.60 (m, 2H), 3.51 (m, 2H), 3.32 (m, 2H), 3.06 (s, 6H), 2.42 (t, J = 7.2 Hz, 2H), 1.77-1.56 (m, 4H), 1.33-1.16 (m, 8H), 0.90-0.78 (m, 3H)

^{13}C NMR (150MHz, D_6-DMSO) δ (ppm): 145.26, 143.38, 132.18, 118.50, 115.86, 115.57, 64.11, 63.88, 62.89, 58.25, 50.68, 32.45, 30.94, 30.68, 25.42, 21.98, 21.74, 13.83

FTIR (cm^{-1}): 3030, 2952, 1599, 1512, 1467, 1380, 1286, 1204, 1036, 966, 811, 765, 633, 537, 492

Z-Cat-C_8 (16)

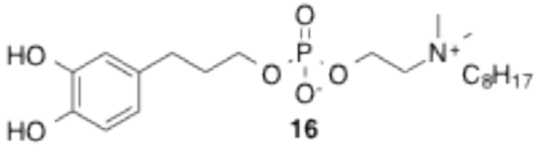

16

Reaction time = 2 days (48 hours) 93% isolated yield.

^{1}H NMR (500MHz, D_6-DMSO) δ (ppm): 9.14-8.43 (s, 1H), 9.14-8.43 (s, 1H) (overlapping), 6.45-6.40 (m, 2H), (dd, *J* = 2, 8 Hz, 1H), 3.85 (m, 2H), 3.48 (q, *J* = 6.5 Hz, 2H), 3.32 (m, 2H), 3.17 (m, 2H), 2.89 (s, 6H), 2.34 (m, 2H), 2.26 (t, *J* = 8 Hz, 2H), 1.58-1.42 (m, 4H), 1.15-1.01 (m, 12H), 0.70 (t, *J* = 7 Hz, 3H)

^{13}C NMR (125MHz, D_6-DMSO) δ (ppm): 145.12, 143.23, 132.37, 118.58, 115.75, 115.44, 64.13, 63.65, 63.60, 63.08, 58.05, 58.01, 50.71, 31.16, 30.96, 28.48, 25.78, 22.03, 21.76, 13.93

FTIR (cm^{-1}): 3029, 2925, 2855, 1599, 1512, 1467, 1378, 1285, 1201, 1061, 1034, 973, 812, 769, 633, 590, 537, 494

MALDI-MS (aCHCA matrix, Low-Res): Calculated for $C_{21}H_{38}NO_6P$: 431.244 Found: 431.308

Z-Cat-C_{10} (17)

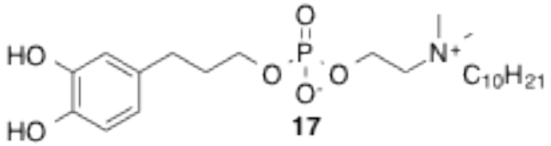

17

Reaction time = 2 days (48 hours) 79% isolated yield.

^{1}H NMR (500MHz, D_6-DMSO) δ (ppm): 9.83-8.55 (br, s, 1H), 9.83-8.55 (br, s, 1H) (overlapping), 6.64 (m, 2H), 6.39 (m, 1H), 4.07 (m, 2H), 3.70 (q, *J* = 6.5 Hz, 2H), 3.52 (m, 2H), 3.36 (m, 2H), 3.06 (s, 6H), 2.43 (t, *J* = 8 Hz, 2H), 1.76 (p, *J* = 7.5 Hz, 2H), 1.67 (m, 2H), 1.32-

1.15 (m, 14H), 0.86 (t, J = 7 Hz, 3H)

^{13}C NMR (125MHz, D_6-DMSO) δ (ppm):141.24, 139.38, 128.12, 114.49, 111.86, 111.57, 60.15, 59.92, 58.89, 54.25, 46.66, 28.44, 27.28, 26.90, 24.93, 24.87, 24.68, 24.56, 21.79, 18.09, 17.81, 9.93

FTIR (cm^{-1}): 3031, 2923, 2854, 1599, 1512, 1467, 1378, 1286, 1204, 1154, 1079, 1037, 967, 812, 770, 634, 591, 538, 492

Z-Cat-C_{12} (18)

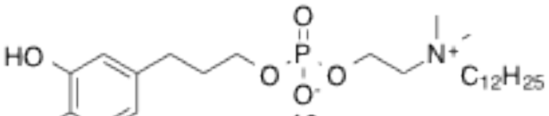

18

Reaction time = 2.5 days (60 hours) 77% isolated yield.

^{1}H NMR (600MHz, D_6-DMSO) δ (ppm): 9.12-8.27 (br, s, 1H), 9.12-8.27 (br, s, 1H) (overlapping), 6.48-6.36 (m, 2H), 6.22 (dd, J = 1.8, 7.8 Hz, 1H), 3.87 (m, 2H), 3.46 (q, J = 6 Hz, 2H), 3.31 (m, 2H), 3.15 (m, 2H), 2.87 (s, 6H), 2.32 (m, 4H), 2.24 (t, J = 7.2 Hz, 2H), 1.56-1.37 (m, 4H), 1.14-0.99 (m, 16H), 0.68 (t, J = 7.2 Hz, 3H)

FTIR (cm^{-1}): 3034, 2922, 2852, 1696, 1599, 1512, 1466, 1444, 1378, 1286, 1202, 1079, 1036, 972, 812, 789, 634, 538, 495

Z-Cat-Cat (19)

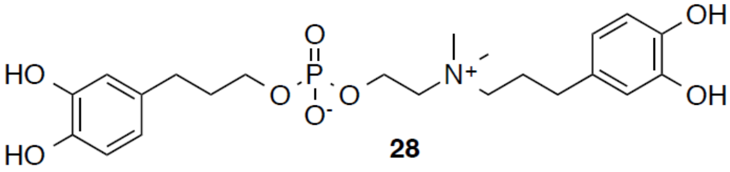

28

Hydrogenolysis of the Z-Cat-Cat-Bn (**18**) was accomplished with a slightly higher catalyst loading (20wt% of Pd/C relative to mass of starting material) and extended reaction time (4 days) for complete de-protection, affording Z-Cat-Cat in 69% isolated yield. ^{1}H-NMR of the product compared to that of starting material showed complete debenzylation. Like it's precursor, Z-Cat-Cat was observed to be highly susceptible to degradation over time, and several unidentified impurities, albeit in low concentration relative to product, were apparent by ^{1}H- and ^{13}C-NMR even when the sample was analyzed within < 30 minutes of isolation. Attempts to increase the level of purity by either recrystallization or HPLC were unsuccessful and the compound was used "as is" for further study. It is recommended that extra care is taken to ensure that this compound be protected from atmospheric oxygen, and be used and analyzed immediately after isolation. Considering that the presence of trace impurities in this case did not give rise to improved or otherwise unexpected performance in adhesieve tests or other false positives and that all other zwitterionic adhesieves were obtained in higher purity, and several displayed higher adhesion, their presence in this case was not seen as problematic as it did not fundamentally affect the interpretation of the data or the broader conclusions of this work.

Reaction time = 4 days (96 hours) 69% isolated yield.

^{1}H NMR (500MHz, D_6-DMSO) δ (ppm): 10.44-8.23 (br, m, 4H), 6.81-6.54 (m, 4H), 6.49-6.34 (m, 2H), 4.04 (m, 1H), 3.75-3.62 (m, 2H), 3.56-3.46 (m, 2H), 3.42-3.36 (m, 1H), 3.35-3.21 (m, 2H), 3.03 (s, 6H), 2.47-2.36 (m, 4H), 1.98-1.86 (m, 2H), 1.77-1.67 (m, 2H).

^{13}C NMR (125MHz, D_6-DMSO) δ (ppm): 145.59, 145.34, 145.10, 143.79, 143.74, 143.24, 132.37, 130.74, 130.22, 118.69, 115.84, 115.78, 115.64, 115.52, 115.37, 65.12, 63.88, 63.32,

58.28, 52.15, 50.79, 30.89, 24.12, 22.95

FTIR (cm^{-1}): 3045, 2954, 1600, 1522, 1473, 1375, 1286, 1196, 1081, 1040, 962, 877, 816, 790

Interfacial and redox characterizations

Aqueous colloidal dispersions of each homolog were prepared in deoxygenated deionized (DI) water. The critical aggregation concentration (CAC) was determined from the solution surface tension of varying homolog concentrations in deionized (DI) water (e.g., see the red line of Z-Cat-C10 in **Figure 2b**) by Wilhelmy plate tensiometry and has been described elsewhere[7]. Use of the CAC instead of CMC here is a hedge to accommodate our uncertainty about whether micelles or other soluble aggregates were forming.

Cyclic voltammetry was performed on a Versastat 3 potentiostat from Ametek Co. These analyses were carried out using a three-electrode cell: a Pt wire as the counter electrode, an Ag/AgCl reference electrode, and a Carbon paste electrode (CPE) served as the working electrode. CPE is the best choice for adhesive materials because of its ability to be completely polished before each experiment. Catechol auto-oxidation was avoided by performing all electrochemical experiments with degassed deionized water in a glove bag under argon. Scan rate 1 mV s^{-1} was used for all experiments. CV of 5 mM synthesized polymers and 5mM methyl catechol, a simple catechol-containing compound, were compared. Oxidation of Catechol carrying molecules occurred at more positive potentials than methyl catechol, indicating the unique structure of the dispersions provide shielding effects that stabilized catechol groups (**Supplementary Figure 1**).

Quartz crystal microbalance with dissipation

Here we briefly explain the general interpretation of the QCM-D data to demonstrate the adsorption of the zwitterionic molecules onto both titania and silica surfaces. Quartz crystal microbalance (QCM) is a surface sensitive technique that measures the change in resonant frequency of a vibrating quartz crystal upon adsorption of material to a surface. The quartz crystal is vibrated by applying a periodic voltage signal across it at its resonant frequency. The resonant frequency of the crystal decreases when the mass of the chip increases (due to adsorption of molecules on its surface), which can be converted to adsorbed mass Δm using the Sauerbrey equation:

$$\Delta m = -\frac{A_c \Delta f \sqrt{\rho_q \mu_q}}{2 f_0^2} \qquad (1)$$

where A_c is the area of the crystal, Δf is the change in frequency, ρq is the density of quartz (2.648 g cm^{-3}), μ_q is the shear modulus of quartz (2.947 × 1011 g cm^{-1} s^2) and f_0 is the resonant frequency of the crystal. The Sauerbrey equation assumes the adsorbed mass is rigid, uniformly distributed across the crystal and the frequency shift is less than 2% of the resonant frequency. Quartz crystal microbalance with dissipation (QCM-D) is an extension to the QCM technique developed by Q-Sense® and can be used to determine the rigidity/softness and the viscoelastic properties of the adsorbed material. The QCM quartz crystal was coated with different rigid materials (e.g., metals, polymers, dielectrics) and the adsorption kinetics can be monitored on these materials in liquid environment. Modeling of the Δf and ΔD at different overtones also allows for the calculation of thin film viscosities, shear modulus, thicknesses, hydrations etc. of the adsorbed layers. Molecule adsorption experiments in a Quartz Crystal Microbalance with Dissipation (QCM-D) indicate that all molecules adsorbed (ΔF) to TiO_2 and SiO_2 surfaces in DI H_2O. The change in dissipation (ΔD) of the adsorbed film shows the degree viscoelasticity for

each film.

Confocal microscopy

An Olympus Fluoview 1000S laser scanning confocal on an inverted microscope stand was used to monitor the intensity change of a polymer tagged with Rhodamine. An uncoated MatTek dish with a #1.5 thick cover-glass bottom was filled with DI water. A 30x silicon objective lens (NA 1.05) and the transmitted light detector were used to focus upon the inner surface of the coverglass surface. Once the polymer was added to the dish, a 559 laser at 4.8% was used to excite the tagged molecules and the confocal PMT detector was used (800x800 pixel, 2 μs pixel^{-1}). A 5 mM solution of Z-Cat-C10 was tagged with ~ 1 mol% Rhodamine dye and applied to a silica surface. The silica surface was then imaged before and after rinsing the surface with DI water. Fig. S6 shows that the molecules stay adheres to the surface and is not washed off unlike the mussel foot proteins that do not spontaneously adhere onto silica surface without a pressure[8]. This shows that the molecules not only adsorb to the silica surface as demonstrated by QCM-D measurements, but also attaches strongly to the surface. Although the dye could perhaps interfere with the adsorption, it is nonpolar, hence the data seem to corroborate the strong adsorption of the zwitterions to silica observed by the SFA, AFM, QCM-D and SEM techniques.

Measuring normal forces in the Surface Forces Apparatus

The interaction between two surfaces can be measured with nano-Newton level force resolution and Angstrom level resolution in the separation distance between the surfaces. The details of the SFA techniques have been describe in a work by Israelachvili et al[9]. In a typical SFA experiment (**Figure 2c**), the distance and the force between the surfaces are measured simultaneously. To begin with, the instrument is calibrated at large separation distances. When the two surfaces are

not interacting, i.e., they are separated by large distances, the change in the separation between them is equal to the distance through which the motor moves the lower surface towards or away from the upper surface (or the upper surface driven by the piezo-tube moves towards the lower surface). However, once the surfaces are close enough to start interacting with each other, the measured separation distance deviates from the expected separation calibrated when there is no force between the surfaces. This deviation is due to the deflection of the double cantilever spring and is directly proportional to the force acting normally between the two opposing surfaces. Thus the normal force can be measured using Hooke's law, $F = k\Delta x$, where k is the spring constant of the double-cantilever spring and $\Delta x = D_{\text{actual}} - D_{\text{applied}}$ is the deflection of the spring, determined by taking the difference between the applied change in position of one of the surfaces D_{applied} and the actual change in distance measured between the surfaces D_{actual}. The actual distance, D_{actual}, between the surfaces can be measured by multiple beam interferometry (MBI) and will be discussed below.

Measuring distance in the SFA by Multiple beam interferometry

The distance between the surfaces, shape of the interface and the refractive index of the media between the surfaces can be accurately determined by Multiple Beam Interferometry (MBI) technique[10]. In this technique, white light is directed through two back-silvered mica surfaces (or uniform and same thickness). As a white light passes between the mica surfaces, it undergoes interference due to the optical trap set up by the back silver on each of these surfaces giving rise to discrete wavelengths of light (**Figure 2c**). These wavelengths of light are resolved in a spectrometer creating interference fringes known as 'fringes of equal chromatic order' (FECO). Since mica is birefringent, the FECO appears as doublets and termed as β and γ. Alternate

fringes are termed as odd and even fringes with odd fringes having nodes at the center and even fringes with anti-nodes in the center. The FECO is then recorded on a camera and analyzed to determine the distance between the surfaces using the following equations:

$$\tan\left(\frac{2\pi\mu_2 D}{\lambda_{\mathrm{n}}^{D}}\right) = \frac{2\bar{\mu}\sin\left(\frac{1-\lambda_{\mathrm{n}}^{0}/\lambda_{\mathrm{n}}^{D}}{1-\lambda_{\mathrm{n}}^{0}/\lambda_{\mathrm{n\text{-}1}}^{0}}\pi\right)}{\left(1+\bar{\mu}^2\right)\cos\left(\frac{1-\lambda_{\mathrm{n}}^{0}/\lambda_{\mathrm{n}}^{D}}{1-\lambda_{\mathrm{n}}^{0}/\lambda_{\mathrm{n\text{-}1}}^{0}}\pi\right)\pm\left(\bar{\mu}^2-1\right)} \quad (2)$$

$$T = n\lambda_{\mathrm{n}}^{0}/4\mu_1 \quad (3)$$

$$n = \frac{\lambda_{\mathrm{n}-1}^{0}}{F_{\mathrm{n}}\left(\lambda_{\mathrm{n}-1}^{0}-\lambda_{\mathrm{n}}^{0}\right)} \quad (4)$$

where D is the separation distance between the surfaces, n is the fringe order (n = 1,2,3,…) λ_{n}^{0} is the wavelength of the nth order fringe (0 refers to the distance between the mica, D = 0, or mica-mica contact reference), T is the thickness of each of the mica surfaces, μ_1 is the refractive index of mica, μ_2 is the refractive index of the medium, $\bar{\mu} = \mu_1/\mu_2$, and the - is used for odd fringes and the + is used for the even fringes, F_{n} is a correction factor that depends on the phase changes at the mica-silver interface and dispersion effects that can be estimated as $F_{\mathrm{n}} \approx 1.024 + 1/\mathrm{n}$ for odd fringes measured near $\lambda \sim 550$ nm[10].

For small separation distance (D < 30 nm) between the surfaces, eq. 2 can be approximated as

$$D = \frac{\lambda_{\mathrm{n}-1}^{0}\left(\lambda_{\mathrm{n}}^{D}-\lambda_{\mathrm{n}}^{0}\right)}{2\mu_1\left(\lambda_{\mathrm{n}-1}^{0}-\lambda_{\mathrm{n}}^{0}\right)}, \text{ for n odd} \quad (5)$$

$$D = \frac{\mu_1\lambda_{\mathrm{n}-1}^{0}\left(\lambda_{\mathrm{n}}^{D}-\lambda_{\mathrm{n}}^{0}\right)}{2\mu_2^2\left(\lambda_{\mathrm{n}-1}^{0}-\lambda_{\mathrm{n}}^{0}\right)}, \text{ for n even} \quad (6)$$

It should be noted that the distance calculated with the equation for the odd fringes (eq. 5) is independent of the refractive index between the two surfaces whereas that calculated with even (eq. 6) is not. This allows for simultaneous measurement of refractive index along with the force and separation distance between the surfaces.

Lap joint bending peel test

The adhesive strength of the synthetic molecules at the macro-scale was demonstrated by gluing two steel plates under water (**Supplementary Figure 12**). Below are the detailed methods following in sequential order in time:

1) The aqueous Z-Cat-C10 solution was prepared at 100 mg ml^{-1} concentration (very turbid solution at room temperature) (**Figure 4a** left).
2) The solution was centrifuged at 3000 rpm (805 × g) for 5min to expedite the process of liquid-liquid phase separation (**Supplementary Figure 12a** middle and right).
3) The lower dense coacervate phase was collected in a syringe (**Supplementary Figure 12a**) and injected (spread, ~10 μl) over a steel plate immersed in water i.e., artificial sea water, aqueous periodate solution, and DI water, respectively (**Supplementary Figure 12b**).
4) Another steel plate glued onto the steel plate with coacervates on as a lap joint (cross-sectional area 2.54 cm × 2.54 cm.
5) The each lap joint was loaded (250 g) for 12h of compressive setting (**Supplementary Figure 12c**).
6) The lap joint bending peel test was conducted *underwater* for each lap joint prepared in artificial seawater, aqueous periodate solution, and DI water, respectively

(**Supplementary Figure 12d-f**).

7) The lap joint bending peel test was conducted *dry* ambient condition for the lap joint prepared in the periodate solution after 12h drying in ambient condition (**Supplementary Figure 12d**).

Standard three point bending peel test

Samples were prepared as described above in "Lap joint bending peel test" underwater, i.e., aqueous periodate solution, then dried 12h and 24h, respectively, in ambient condition. In this standard three point bending peel test, the cross section area was 2.54 cm × 1.27 cm.

The standard three point bending peel test (**Figure 3d**) was conducted with a Bionix 200 tensile tester (MTS Systems) in ambient conditions. All tests were conducted with a test speed of 5 mm min^{-1}.

The catecholic zwitterions were inspired by interfacial proteins (mfp-3 and -5) that function as adhesive primers, but not as bulk adhesives. To determine whether Z-Cat-C10 is limited to surface binding or develops measurable bulk cohesion (thickness <10 μm), bulk rupture tests were done using a peeling-by-bending test to better mimic the role that interfacial proteins play in resisting plaque detachment as well as lap-shear bonding test. Lap joints were prepared by gluing two steel plates (SS304) immersed in water using the lower dense coacervate (~10 μl) (**Supplementary Figure 12** and **Supplementary Movie 3**). The lap joint (cross-sectional area=2.54 cm × 2.54 cm) prepared in artificial seawater or periodate solution, respectively, held a load up to 3 N underwater. On the other hand, the lap joint prepared in DI water held only 1 N, suggesting that oxidative cross-linking of catechols is necessary to obtain high adhesion as shown in the SFA study described below. After 12 h ambient drying, the joint

prepared in 1 M periodate solution held much higher load, 11 N. Standard three point bending peel strength of the lap joint (cross-sectional area=2.54 cm × 1.27 cm) was measured under ambient dry conditions after drying 12 h. The peel strength and the lap shear bonding strength of the joint made with Z-Cat-C10 in the periodate solution exhibited 20.5 N cm^{-1} (standard deviation=0.9, n=4) — stronger than the joint with 3M double sided Scotch tape®, 12.0 N cm^{-1} (standard deviation=0.1, n=4), prepared under ambient dry conditions. Shear bonding strength of the lap joint (cross-sectional area= 2.54 cm × 2.54 cm) was also measured, 1.1 MPa (standard deviation= 0.3, n=4) — similar to Dopa-functionalized polypeptide prepared under ambient dry conditions, 0.9 MPa[6]. The bulk test results shown here did not overwhelm the previously reported bulk adhesives, but it was expected because the small molecular surface primer is not a bulk adhesives. Nevertheless, the results present that the zwitterionic surfactants were capable of micro-gap-filling as well as nano-gap-filling shown in AFM and SFA, and indicate that they are more versatile than surface primers. The results also attest to the self-assembling ability of Z-Cat-C10 (Figure 4a-c, double bilayer structure).

X-ray photon spectroscopy (XPS)

Kratos Axis Ultra (Kratos Analytical, Manchester UK), was conducted with survey scans at a pass energy of 160 eV, and high-resolution scans at a pass energy of 40 eV. XPS of copper plate (**Supplementary Figure 21-23**) showed the copper surface contains ~ 60:40 oxide:hydroxide. However, silicon wafer (**Supplementary Figure 24-26**) has only oxide. The hydroxide is thought to weaken the adhesion to copper plate in AFM compared to silicon wafer in addition to its higher RMS roughness.

Nano layer adhesion to silica surface

Silica beads (diameter φ = 100 nm) dispersion (10 mg ml^{-1} in DI water) was spread on a silicon wafer coated with Z-Cat-C10 described above. The glued beads were, then, rinsed thoroughly with DI water. The resulting surface was then imaged with SEM to determine if the beads adhered to the Z-Cat-C10 coated silicon wafer surface. The beads were maintained to adhere onto the silicon wafer surface (**Fig. 4e**) whereas there is no silica bead on the bare silicon wafer surface. The strength of adhesion and film dimensions of catechol hold particular promise for nano-length scale tailoring[11] that obviate current thicker adhesion promoters, e.g., high performance nanometer thin adhesives to enhance the power delivery and energy storage capacity of Li-ion batteries with high density of silicon nanoparticles by replacing the thick polymer adhesive binders currently used in Li-ion battery anodes[12].

Supplementary References

1 Peresypkin, A. V. & Menger, F. M. Zwitterionic geminis. Coacervate formation from a single organic compound. *Org. Lett.* **1**, 1347-1350 (1999).

2 Panne, P. & Fox, J. M. Rh-catalyzed intermolecular reactions of alkynes with α-diazoesters that possess β-hydrogens: ligand-based control over divergent pathways. *J. Am. Chem. Soc.* **129**, 22-23 (2007).

3 Fulmer, G. R. *et al.* NMR chemical shifts of trace impurities: common laboratory solvents, organics, and gases in deuterated solvents relevant to the organometallic chemist. *Organometallics* **29**, 2176-2179 (2010).

4 García, G. *et al.* Losartan-antioxidant hybrids: novel molecules for the prevention of hypertension-induced cardiovascular damage. *J. Med. Chem.* **52**, 7220-7227 (2009).

5 García, G. *et al.* New losartan-hydrocaffeic acid hybrids as antihypertensive-antioxidant dual drugs: Ester, amide and amine linkers. *Eur. J. Med. Chem.* **50**, 90-101 (2012).

6 Thanh, T. & Chabrier, P. New method of preparation for phosphorylcholine, phosphorylhomocholine and their derivatives. *Bull. Soc. Chem. Fr.* **3**, 667-671 (1974).

7 Das, S., Donaldson Jr, S. H., Kaufman, Y. & Israelachvili, J. N. Interaction of adsorbed polymers with supported cationic bilayers. *RSC Adv.* **3**, 20405-20411 (2013).

8 Akdogan, Y. *et al.* Intrinsic Surface-Drying Properties of Bioadhesive Proteins. *Angew. Chem., Int. Ed.* **53**, 11253-11256 (2014).

9 Israelachvili, J. *et al.* Recent advances in the surface forces apparatus (SFA) technique. *Rep Prog Phys* **73**, (2010).

10 Israelachvili, J. Thin-Film Studies Using Multiple-Beam Interferometry. *J. Colloid Interf. Sci.* **44**, 259-272 (1973).

11 Gandhi, D. D. *et al.* Annealing-induced interfacial toughening using a molecular nanolayer. *Nature* **447**, 299-302 (2007).

12 Magasinski, A. *et al.* High-performance lithium-ion anodes using a hierarchical bottom-up approach. *Nat. Mater.* **9**, 353-358 (2010).
